# Experimental platform for the functional investigation of membrane proteins in giant unilamellar vesicles†

**DOI:** 10.1039/d2sm00551d

**Published:** 2022-07-25

**Authors:** Nicolas Dolder, Philipp Müller, Christoph von Ballmoos

**Affiliations:** Department of Chemistry, Biochemistry and Pharmaceutical Sciences, University of Bern Freiestrasse 3 3012 Bern Switzerland christoph.vonballmoos@unibe.ch

## Abstract

Giant unilamellar vesicles (GUVs) are micrometer-sized model membrane systems that can be viewed directly under the microscope. They serve as scaffolds for the bottom-up creation of synthetic cells, targeted drug delivery and have been widely used to study membrane related phenomena *in vitro*. GUVs are also of interest for the functional investigation of membrane proteins that carry out many key cellular functions. A major hurdle to a wider application of GUVs in this field is the diversity of existing protocols that are optimized for individual proteins. Here, we compare PVA assisted and electroformation techniques for GUV formation under physiologically relevant conditions, and analyze the effect of immobilization on vesicle structure and membrane tightness towards small substrates and protons. There, differences in terms of yield, size, and leakage of GUVs produced by PVA assisted swelling and electroformation were found, dependent on salt and buffer composition. Using fusion of oppositely charged membranes to reconstitute a model membrane protein, we find that empty vesicles and proteoliposomes show similar fusion behavior, which allows for a rapid estimation of protein incorporation using fluorescent lipids.

## Abstract 2

Relevant steps of functional membrane protein investigation in giant unilamellar vesicles (GUVs) are compared for GUVs formed by PVA assisted swelling or electroformation under physiologically relevant conditions.

## Graphical abstract

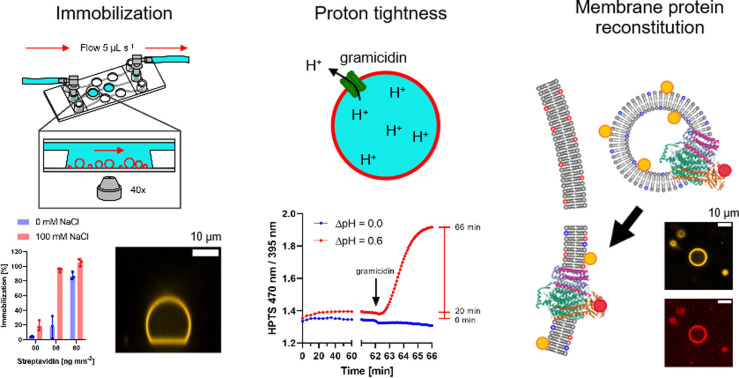

## Introduction

Giant unilamellar vesicles (GUVs) are micrometer-sized (1–100 μm) model membrane systems serving for *in vitro* studies of various lipid membrane related processes, such as membrane fusion and fission, cell division or lipid domain formation.^1^ GUVs are also considered as scaffolds for the bottom-up creation of synthetic cells^2–4^ and the targeted delivery of drugs.^5^ Another, much less explored application of GUVs is the investigation of membrane proteins (MPs) that are responsible for many cellular key functions, such as nutrient uptake, signal transduction, energy metabolism and regulation of the structure and dynamics of the membrane.^6^ In humans, altered expression or activity of MPs is related to many diseases, such as neurodegenerative disorders, diabetes and certain cancers.^7–10^ Being accessible on the cellular surface, MPs are considered excellent drug targets.^11^ A profound understanding of the enzyme mechanism and a robust platform to investigate mechanism and potential drug targets are key to the successful development of new treatment methods. With their size close to eukaryotic cells that reduces curvature stress compared to small vesicles, GUVs are an attractive platform for MP analysis. Since measurements are possible on a single vesicle level, much less protein is required, opening this field also to poorly expressing human proteins.

In our laboratory, we are investigating the molecular mechanism of different MPs, *e.g.* solute transporters and members of the respiratory chain from bacteria and eukaryotes.^12–14^ As others, we were attracted by the unique possibility of GUVs to follow the function of our proteins of interest, often including the transport of substrates as small as protons, directly under the microscope. The first functional reconstitution of a MP into GUVs has been described already twenty years ago, but until today only around a dozen publications demonstrate convincing light microscopic measurements with such transporters.^15–25^ Although these publications impressively show the potential of the methods, applications beyond proof-of principle are rare. While the need for suitable detection systems makes these measurements inherently challenging, fluorescent reporters for protons and other ions such as sodium are well established and enable the indirect detection of the activity of many secondary active transporters as well.^26^

The first step to perform such measurements is the efficient formation of GUVs. Vesicles should be produced in a suitable size range and yield that a sufficient number of free-standing GUVs can be identified as unilamellar vesicles in the field of view of the microscope. Very large vesicles are further undesirable due to concerns with stability and tightness of the membrane. A diameter of 5–20 μm, compatible with the size range of many eukaryotic cells, is considered to be well suited for our applications, allowing for parallel observation of >20 vesicles in the 40×-field of view. This number ensures that different parameters such as protein density, vesicle size and shape are represented in sufficient diversity that the experiment reproduces a meaningful picture of the observed reaction. Formation should further take place under physiologically relevant conditions, including relatively high salt and buffer concentrations that stabilize MPs and are used to generate electric Nernst potentials. Generally, solvent free methods would be preferred as traces thereof in the membrane (*e.g.* within the hydrophobic part of the bilayer) affects membrane thickness and is likely to negatively affect MP function. Details on various GUV formation methods have been extensively described, although quantitative comparisons are rare.^27–30^ To understand further difficulties of these experiments and to give a broader perspective on the challenges of MP investigation in GUVs, our described experiments also include the experimental steps after GUV formation, *i.e.* GUV immobilization, proton leakage, and protein reconstitution.

Immobilization of GUVs on the microscopy slide is not only desirable for the long-term observation of single vesicles to follow the function of MPs inside a GUV, but it is critical if reagents have to be added during the process. Immobilization has been achieved using various strategies, both physical^31–35^ and non-specific^34,36–38^ or specific chemical interactions.^36^ Due to its high specificity and affinity (*K*_D_ ∼ 10^−15^ M), the biotin-avidin interaction has become a popular immobilization strategy.^38,39^ However, it has been shown that strong vesicle adhesion forms a spherical cap on the surface^40^ that can lead to an increase in membrane tension and formation of pores, making GUVs prone to leakage, or even cause rupture of the vesicles.^40–42^ Such leakage is detrimental for measuring vectorial substrate transport or if an electrochemical proton gradient is involved, such as in secondary transporters or respiratory enzymes. In general, relatively little data is available regarding the tightness of the GUV membrane towards certain substrates,^43,44^ especially small ones such as protons.^24^

Functional reconstitution of the MP into the GUV membrane is critical for all downstream applications. Several methods including detergent-mediated reconstitution, rehydration of partially dehydrated SUVs or fusion of SUVs to GUVs have been exploited for MP reconstitution in GUVs.^45^ We and others have recently used charge-mediated fusion to introduce several MPs into GUVs, including multisubunit alpha-helical enzymes, indicating a general applicability of the method.^46–48^ This reconstitution strategy is a mild approach that transfers one or more MPs from SUVs containing positively charged lipids (reconstituted with traditional methods) to GUVs containing negatively charged lipids,^46,47^ preserving the protein orientation.^46^ This fusion strategy is not without concerns, and its limitation are discussed later.

Here, using polymer assisted swelling^49–52^ and electroformation^53^ methods, we compare the produced GUVs on vesicle properties important for investigating MPs in GUVs as outlined above. We quantify the ability of these formation methods to produce a high number of vesicles with diameters between 5–20 μm under physiologically relevant conditions and characterize whether immobilization using the biotin-streptavidin system was affected by the presence of salt. Vesicle leakage is analyzed using influx measurements of the hydrophilic dye pyranine (HPTS). GUVs that did not show HPTS leakage were further analyzed using a proton efflux assay to assess the ability of GUVs to maintain a pH gradient. As a last step, we used fluorescently labeled cytochrome *bo*_3_ ubiquinol oxidase as a model protein to monitor incorporation *via* charge mediated fusion.

## Results

### GUV formation

The best-known method for GUV formation is based on electroformation, in which hydration of dried lipids is aided by an oscillating electrical field with different frequencies and voltages. More recently, polymer assisted swelling was reported as a simple and versatile alternative and a variety of polymers have been used as substrate.^49–52,54,55^ Especially, production of GUVs with various lipid and buffer compositions has been demonstrated without the need for optimizing the method.^49–52^ This is in contrast to electroformation where protocols typically must be adapted, *i.e.* to form GUVs in buffers containing physiological salt concentrations^51,56–58^ or using cationic lipids.^59^ This makes polymer assisted swelling a desirable method for the investigation of MPs as variation of the lipid and buffer composition are common requirements to obtain optimal enzyme activity or to investigate lipid effects. Among the different substrates used, polyvinyl alcohol (PVA) has established itself as a go-to material due to its wide accessibility and ease of use. However, the method is not without pitfalls, which have been explored in several previous publications,^50,60,61^ such as for example contamination of the lipid bilayer with residual PVA.^60^ Despite this, we have focused on the use of PVA and compared GUV properties important for MP investigation for vesicles produced by PVA assisted swelling and electroformation. As a basis for this comparison, we deemed a quantitative formation analysis necessary under desired conditions. Here, we focused on a lipid composition of 70% DOPC and 30% DOPG for two main reasons. First, native membranes, *i.e.* bacterial or mitochondrial inner membrane, show an overall negative charge of about 30% with PC and PG being main constituents. Second, in our experience with charge mediated fusion, 30% negatively charged lipids have been a good compromise for fast and complete fusion.^46^ Variation of the lipid composition during GUV formation has been successfully described,^50,61^ and is unlikely to cause problems in similar experiments.

Our focus was the number of GUVs with diameters between 5 and 20 μm that we considered suitable for our purposes (see above). GUV formations were considered as not successful if <10 GUVs in the desired size range were observed per field of view under the same experimental formation conditions. We considered 1.5 × 10^5^ GUVs per mL in the requested size range as sufficient. The number is based on the observation that loading of 100 μL vesicle suspension should lead to at least 20 GUVs per field of view. A detailed description of the quantitative analysis is presented in the ESI† (Fig. S3). Briefly, in the absence of salt, all three formation methods (PVA assisted swelling, Pt wire and ITO electroformation) produced sufficient concentration of GUVs (>7 × 10^5^ mL^−1^). In the presence of salt, only PVA and Pt wire formation yielded a suitable number of GUVs, due to limitations in our ITO electroformation setup.^58^ Generally, GUV concentration was reduced with salt in the buffer solution. In all cases, PVA formation resulted in the highest concentration of GUVs. In agreement with previous studies, we observed an increase in vesicle size for Pt wire formation in the presence of salt,^57^ leading to an increased fraction of GUVs in the desired size range. In contrast, increased salinity did not result in a higher fraction for PVA formation.

### GUV immobilization

**Fig. 1:**
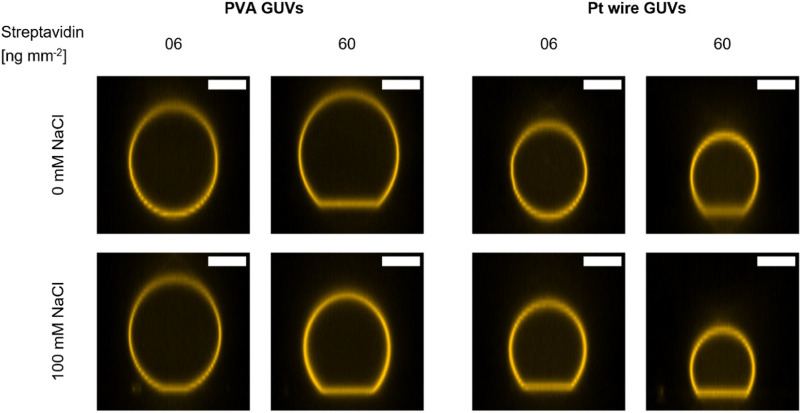
Formation of spherical adhesion cap at low and high streptavidin density. PVA and Pt wire GUVs in the presence and absence of 100 mM NaCl were immobilized at different streptavidin densities and confocal Z-stacks were recorded. Side-views of representative GUVs from each condition are shown. Spherical caps are observed for all conditions at high streptavidin densities while low densities show no or few caps in the absence of NaCl. The scale bar is 10 μm.

**Fig. 2:**
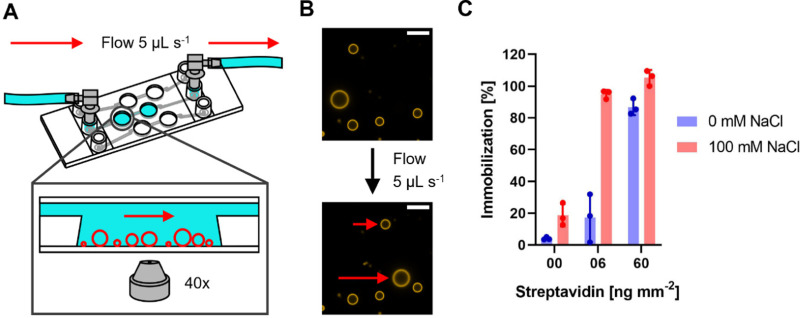
Immobilization assay using channel slides and PVA GUVs. (A) Schematic representation of the channel slide setup with a blow-up depicting the immobilized GUVs (red circles) in the well of the channel. (B) Example of immobilized GUVs under flow. Top image depicts GUVs before and bottom image during application of flow. Non-immobilized GUVs are indicated by the red arrows. The scale bar is 10 μm. (C) Percentage of immobilized PVA GUVs in the presence or absence of 100 mM NaCl at different streptavidin densities assessed by comparing the number of immobilized GUVs and the number of GUVs before application of flow. Data from three independent experiments are shown. 40–120 GUVs were counted for analysis. The height of the bar indicates the average percentage of immobilization with individual values from the experiments shown as dots. Error bars indicate the standard deviation.

Typically, spectroscopic assays for MPs reconstituted in SUVs last from a few seconds to several minutes. Often, at one or more timepoints, substrates or other chemicals are added to start the reaction or otherwise influence it. It is therefore indispensable to immobilize GUVs to observe them in the same field of view over a prolonged time. Here, we focus on the well-known interaction between biotin and streptavidin that is frequently used as an immobilization system,^62,63^ in which biotinylated lipids in the GUV membrane interact with the streptavidin coated surface of a microscopy slide. The system allows to fine-tune the degree of immobilization by varying the concentration of either biotinylated lipids or streptavidin. We anticipate that also the ionic strength of the surrounding buffer might influence the immobilization behavior of GUVs. To standardize the immobilization condition between different types of microscopy slides, we calculated the streptavidin amount per area of slide surface in contact with the streptavidin solution, and the value was termed “streptavidin density” in [ng mm^−2^] and is used from now on. In the next series of experiments, GUVs produced *via* the PVA and Pt wire method were immobilized using two different streptavidin densities, both in the presence and absence of 100 mM NaCl. As shown in Fig. 1, a spherical adhesion cap was observed for both formation methods at high streptavidin density, and the presence of NaCl promoted the formation of adhesion caps at low streptavidin density. No caps were observed without streptavidin (Fig. S6A, ESI†) in either condition. Differences in immobilization depending on the buffer composition are rarely reported in studies using GUVs for light microscopic MP measurements, therefore we wanted to quantitatively analyze the differences observed in Fig. 1. Since cap sizes within one condition were rather heterogenous (Fig. S6B, ESI†), quantification of immobilization was not straightforward. Using commercial channel slides, we developed a flow-based assay that greatly facilitated quantification of the immobilization behavior (Fig. 2A). Since PVA and Pt wire GUVs showed comparable immobilization in 8 well chambered slides (Fig. 1), only PVA GUVs were used in flow experiments due to the higher formation yield.

In this assay, PVA formed GUVs were immobilized at different streptavidin densities in presence and absence of 100 mM NaCl. First, all GUVs in the field of view were counted, before a constant flow of 5 μL s^−1^ was applied and the number of GUVs which withstood the flow were counted to determine the immobilization percentage. The general trend of the 8 well slide experiments was also observed in the flow-based immobilization assay. Without streptavidin, no immobilization was observed and at high streptavidin density almost all observed GUVs were fully immobilized in presence as well as in absence of NaCl (Fig. 2C). However, GUVs immobilized at 6 ng mm^−2^ streptavidin in absence of NaCl showed almost no immobilization, whereas GUVs immobilized at the same streptavidin density but in presence of NaCl where immobilized almost to 100%. This indicates that immobilization using the biotin streptavidin system is highly salt sensitive.

### HPTS and proton leakage

**Fig. 3:**
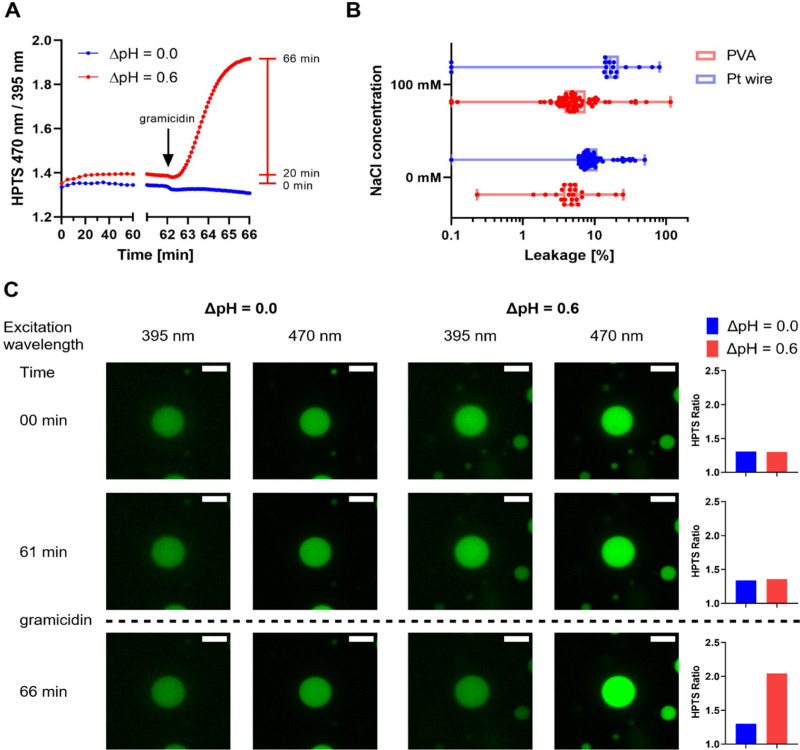
Proton leakage of immobilized PVA and Pt wire GUVs in the presence or absence of 100 mM NaCl. Analysis was limited to GUVs with diameters of 5–20 μm in the focal plane. Data are taken from a single time series for each condition. (A) Average HPTS ratio of PVA GUVs with 100 mM NaCl subjected to a pH gradient of 0.0 and 0.6 over time (pH inside = 7.4, pH outside = 8.0). After 62 min, gramicidin was added to equilibrate the inner and outer pH, leading to an efflux of protons and an increase in HPTS ratio if a pH gradient is applied while no change is observed without pH gradient. The red bar indicates the time points used to assess the percentage of leakage. (B) Box plot showing the percentage of leakage of PVA (red) and Pt wire GUVs (blue) with individual GUVs indicated as dots. Proton leakage was calculated by dividing the increase of the HPTS ratio after 20 min by the total increase from 0 min to 66 min (after addition of gramicidin). 20–70 GUVs per field of view were considered for analysis. (C) Confocal microscopy images of an exemplary PVA GUV with 100 mM NaCl from the measurement with or without a pH gradient shown in (A). The two HPTS channels (green) are shown at different time points during the experiment. GUVs are shown at the start (00 min), after 1 h incubation before (61 min) and after (66 min) the addition of gramicidin. On the right, the HTPS ratio of the GUVs depicted on the left were calculated. The scale bar is 10 μm. All images were processed identically.

To observe whether strong adhesion at high streptavidin densities affects the integrity of immobilized GUVs, we performed a dye permeation assay using HPTS. Empty GUVs were allowed to immobilize in the presence of HPTS on the outside and dye leakage into vesicles was monitored. At low streptavidin concentrations, almost no leakage was observed, while the fraction of leaky GUV increased with increasing streptavidin concentration, but never exceeded 35% (Fig. S7 and S9, ESI†). In addition, slight differences were observed for PVA and Pt wire GUVs. For PVA GUVs, the optimal streptavidin density was found to be between 6 and 30 ng mm^−2^ and PVA GUVs in the presence of salt remained leaky after immobilization (Fig. S8 and S9, ESI†). The results are discussed in more detail in the ESI.† HPTS has a molecular weight of 524 g mol^−1^, and thus is larger than many biological substrates of interest. Protons, the smallest biological unit, are of special interest in our research with respiratory enzymes and we therefore extended our leakiness tests with GUVs to protons using a gramicidin assay (Fig. 3). Here, GUVs containing the ratiometric proton sensitive fluorophore HPTS,^64–66^ which reports pH changes in the GUV lumen, are subjected to a proton gradient. Using a ratiometric dye allows for easy correction of bleaching effects. An increase of the pH in the GUV lumen leads to an increase in the ratio of the two HTPS signals. To increase the sensitivity of the assay we lowered the amount of buffering molecules by adjusting MOPS buffer to the desired pH with KOH instead of bis-tris propane (BTP). Surprisingly, PVA GUVs prepared in 5 mM MOPS-KOH in the absence of salt showed insufficient immobilization even at high streptavidin densities (Fig. S10, ESI†), but immobilization was possible using 10 mM MOPS-KOH and 10 ng mm^−2^ streptavidin. To detect proton leakage, GUVs containing HPTS were prepared at pH 7.4, immobilized and washed with buffer at pH 8.0, leading to a gradient of 0.6 (inside acidic). The HPTS signal was monitored for 60 min before the pH gradient was equilibrated by the addition of the protonophore gramicidin, leading to an alkalinization of the GUV lumen and an increase in HPTS ratio (Fig. S11, ESI†). No change in signal was observed after gramicidin addition in the absence of a pH gradient, indicating that the change in signal in presence of the pH gradient corresponds to the alkalinization of the GUV lumen. (Fig. 3A, C and Fig. S11E, and F, ESI†).

Experiments with MPs are unlikely to last more than one hour. Thus, we analyzed only the increase in HPTS ratio in the first 20 min and compared them to the total increase after addition of gramicidin (Fig. 3A). A small number of GUVs showed a decrease in HPTS ratio in the first 20 min and were excluded from the analysis. Decreasing signals might be observed in GUVs with HPTS leakage before or during incubation or due to microscope drift. With the remaining GUVs, the percentage of the increase after 20 min was calculated, assuming a total pH equilibration after gramicidin addition (Fig. 3B). Most GUVs that were analyzed showed very little increase after 20 min. In those, proton leakage of less than 20% in the first 20 min was generally observed, indicating that the vesicles are sufficiently tight towards proton efflux if HPTS had not leaked. A slightly lower percentage of leaky GUVs was found in the PVA than in the Pt wire preparation, and no influence of salt on leakage was observed in either of the preparations. A more detailed discussion of the data is found in the ESI.†

### Membrane protein reconstitution

**Fig. 4:**
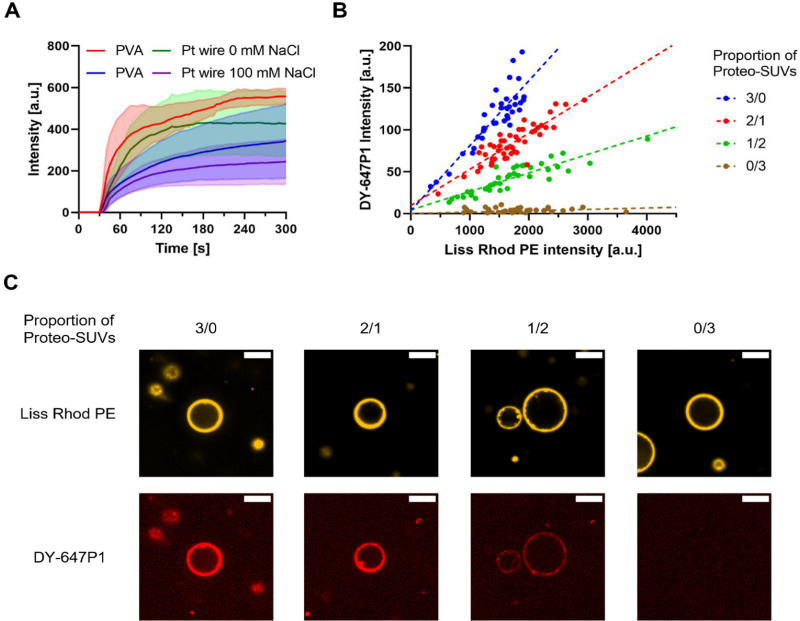
Charge mediated fusion of positively charged SUVs with negatively charged GUVs. Fusion was performed in an 8 well chambered slide with addition of SUVs to immobilized GUVs. Only GUVs with diameters of 5–20 μm in the focal plane were analyzed. (A) Fusion of GUVs with empty SUVs (final concentration 10 μg mL^−1^) followed in real time. SUVs were added to the well after 30 s after which an increase of Liss Rhod PE signal in the GUV membrane was observed. Traces represent the mean (solid line) and standard deviation (transparent area above and below the trace) of the average intensity increase of 10–50 GUVs from 4 experiments. (B) Fusion of PVA GUVs at 0 mM NaCl with empty SUVs and proteo-SUVs (final concentration 40 μg mL^−1^) containing DY-647P1-labeled cytochrome *bo*_3_ ubiquinol oxidase. Empty and proteo-SUVs were mixed at different ratios with proteo-SUV proportions of 3/0, 2/1, 1/2 and 0/3 v/v. The DY-647P1 intensity is compared to the Liss Rhod PE intensity 150 s after addition of SUVs. A similar distribution of Liss Rhod PE intensities is observed in all fusion reactions as both empty and proteo-SUVs contain Liss Rhod PE. The DY-647P1 shows a linear dependency to the Liss Rhod signal with a decreasing slope as the proportion of proteo-SUVs decreases. Values of individual GUVs from one experiment are shown (30–50 GUVs) as dots and the linear regression is represented as a dotted line. (C) Confocal microscopy images of representative GUVs from fusion with different proteo-SUV proportions shown in (B). For each GUV, the Liss Rhod PE and DY-647P1 channel are depicted. The scale bar is 10 μm. All images from each channel were processed identically.

The overall negative surface charge of lipid composition of PC : PG (7 : 3) not only reflects conditions found in biological membranes, but is also compatible with fusion of oppositely charged proteo-SUVs.^46–48^ Here, the MP of interest can be individually reconstituted into SUVs containing positively charged lipids under optimal conditions and possibly with a desired orientation.^3^ If these SUVs are mixed with negatively charged GUVs, membrane fusion occurs and the orientation of the membrane protein is conserved.^3,46^ Smaller SUVs have been demonstrated to be more efficient in these experiments,^46^ and SUVs formed by sonication (25–50 nm diameter)^67^ have been used here. This modular approach is ideally suited for the bottom-up construction of artificial cells that contain several different MPs that have non-compatible reconstitution procedures. The reconstitution yield can be directly monitored, if the MP is fluorescently labeled itself. However, this is not always feasible (impaired activity after labeling, low amount of protein, no suitable labeling chemistry). We therefore hypothesized that protein reconstitution can be correlated with fusion efficiency (which can be followed by fluorescent lipids). We were also interested to see if fusion of oppositely charged vesicles is dependent on the salt concentration, a topic that has been discussed with some controversy in the literature.^46,47,68^ To tackle the latter question, we thus investigated charge mediated fusion using protein free SUVs, prepared using sonication and containing a lipid coupled fluorescent dye, with unlabeled GUVs in presence and absence of salt. With this approach, fusion was quantified by the amount of lipid coupled dye found in the previously unlabeled GUV membrane. If experiments were performed directly under the microscope using immobilized GUVs, rapid fusion is observed (movie S1, ESI†) with positively charged SUVs while no fusion was observed with neutral SUVs (Fig. S12A, ESI†). GUVs were fusogenic both in presence and absence of salt, although a smaller fluorescence increase in the GUV membrane was observed in the presence of salt (Fig. 4A and Fig. S12B, ESI†). The opposite charges of the lipids may be screened by the presence of salt, thereby decreasing the fusion efficiency. However, the signal distribution in the raw data was rather heterogenous and we were not convinced of a significant influence of salt on the fusion process (Fig. S13 and S14A, ESI†). To eliminate effects of immobilization and mixing or pipetting artefacts during addition under the microscope, we repeated the experiments by mixing SUVs and non-immobilized GUVs in an Eppendorf tube that were deposited on a microscopic slide without immobilization (Fig. S14B and S15, ESI†). While PVA GUVs still show a slightly decreased fusion behavior in presence of salt, this trend was not observed with Pt wire GUVs (Fig. S14B, ESI†). This difference in observed fusion behavior of Pt wire GUVs either immobilized on slides or in solution is not straightforward to explain. Additions to immobilized GUVs are performed with utmost care but mixing and diffusion of added chemicals are likely to differ from experiment to experiment that might lead to a non-homogenous distribution of SUVs upon addition in a single experiment. A detailed description is given in the ESI.† Nevertheless, GUVs appeared fusogenic under all conditions.

Finally, we investigated fusion of proteoliposomes containing rhodamine-labeled lipids and DY-647P1-labeled *bo*_3_ oxidase in the absence of salt with PVA GUVs that showed a slightly higher fusion yield in the previous experiment (Fig. 4B, C and Fig. S16, ESI†). Prior to fusion, proteoliposomes (protein and lipid labeled) and empty liposomes (only lipid labeled) were premixed in different ratios (3/0, 2/1, 1/2 and 0/3, *v*/*v*) to simulate the insertion of different amounts of enzymes. Of this mixture, a SUV to GUV lipid molar ratio of ∼160 was added to immobilized GUVs. As depicted in Fig. 4B, a linear correlation between *bo*_3_ oxidase signal and lipid-coupled dye was observed in the different experiments. This is a strong indication that empty SUVs and enzyme containing vesicles have the same fusion properties and that the intensity of lipid-coupled dye correlates with the intensity of labeled *bo*_3_ oxidase and can be used to estimate the relative amount of enzyme reconstituted.

## Discussion

GUVs are attractive model systems for a variety of biological questions, including the investigation of membrane proteins, but a broad application for the latter topic has not been taken place. In our lab, we are investigating different membrane proteins with substrates as small as proton or sodium ions, requiring robust and tight vesicles, also in the presence of physiological salt concentrations. As additions of substrates during the measurements are required, GUVs need to be immobilized to be monitored over a prolonged time. Reconstitution yield of membrane proteins into GUVs should be predictable to compare activities from single GUVs allowing to divide them into subpopulations. All these factors are highly important if GUVs should serve as a versatile and powerful model system to functionally characterize a MP beyond binding studies and serving as a spherical container.

Here, we compared the popular electroformation method with the more recently developed polymer assisted swelling for GUV formation in the presence of mono and -divalent ions. Having their application for membrane proteins in mind, we used a lipid composition of 70% DOPC and 30% DOPG. A overall negative surface charge of ∼30% is in good agreement with many cellular membranes and with popular lipid mixtures used for membrane protein studies in small and large unilamellar liposomes (*e.g. Escherichia coli* polar lipid extract or soybean extract). For electroformation, either Pt wires or ITO coated glass slides were used. PVA assisted swelling and electroformation have been compared in the literature^51,69,70^ and the present data extend this knowledge with our comprehensive quantitative analysis using conditions for biological questions. Overall, PVA assisted GUV formation in the presence of salt tended to decrease the concentration of vesicles. This is in agreement with previous results for agarose assisted swelling where an increasing ionic strength led to a decrease in vesicle concentration independent of lipid charge.^71^ Similar results were observed for polyacrylamide assisted swelling, although formation was worst in pure water and best at 100 mM sucrose.^52^ In contrast, few polymer substrates even showed increasing GUV concentrations with increasing ionic strength or osmolarity.^72^ Taken together, the impact of ionic strength on the GUV formation process seems unaffected by the lipid composition, but may depend on the swelling-assisting substrate which is used. Only a slight decrease in vesicle concentration was observed in Pt wire electroformation experiments, while the lipid yield was mostly unaffected due to an increase in vesicle diameter, as was previously observed for electroformation using up to 100 mM NaCl.^57^ A decreased influence of salt on Pt wire formation may be observed due to an electric-field dependant lipid bilayer destabilization, favoring GUV formation, which is in competition with hindered lamellae separation in presence of salt.^57^

The immobilization behavior of PVA and Pt wire GUVs was investigated using the widely used biotin streptavidin system in presence and absence of salt. Strong adhesion of vesicles to a slide surface has been shown to results in the formation of a spherical adhesion cap.^38,40^ Here, we found that cap formation depends on the salinity of the buffer as well as the amount of streptavidin used for immobilization. Previously, modulation of the adhesion cap size was demonstrated by adjusting the MgCl_2_ concentration in the medium.^73^ Our results and others indicate that this is likely true for other ions as well.^74^ Adhesion cap formation was strictly dependent on the presence of streptavidin, as no unspecific binding was observed on BSA coated slides. In our flow-based assay, we find that at the lowest tested streptavidin density of 6 ng mm^−2^, 20% and 100% of GUVs were immobilized in the absence or presence of NaCl, respectively. We also found an influence of the counterion (KOH or BTP) of the buffer (MOPS) if no salt was used, and that a higher buffer concentration improved immobilization. Vesicle attachment to the slide surface is determined by the competition between the adhesive strength (that is the membrane-substrate interaction) and the bending rigidity of the vesicle membrane, the former one being sufficiently large or the latter one sufficiently small.^1^ The presence of salt or other charged molecules in the buffer might influence the membrane-surface interaction, *e.g.* by shielding repulsive charges or bridging charged species on the surface and in the lipid membrane. It has been shown that salt facilitates the formation of supported lipid bilayers which is mediated by vesicle adhesion on glass support.^74^ Both buffers and ions have also been shown to affect the bending rigidity of the membrane,^75,76^ but effects depend on the membrane composition.^77^ Finally, the interaction of biotin and streptavidin has been shown to increase with increasing salinity of the buffer system.^78,79^ In one report, a threefold increase of biotinylated DNA binding to streptavidin coated beads was observed in the presence of 100 mM NaCl.^78^ In our setup, it is difficult to discern between improved biotin streptavidin interaction or increased unspecific interaction of the membrane with the biotin-BSA coated surface. Only the latter, however can be responsible for the slight increase of immobilized GUVs observed in the absence of streptavidin, but in presence of 100 mM NaCl.

Strong adhesion is known to trigger leakage through water permeation or through the formation of pores, at least temporarily to adjust for the shape changes induced by vesicle attachment.^40,41^ In agreement, strong adhesion has been shown to increase the membrane tension in GUVs^73,80^ with tension being a potential driving force for the formation of transient pores.^42^ GUV content leakage must be avoided for measuring MPs with vectorial transport functions and will also lead to inhomogeneous concentrations of encapsulated dyes, complicating quantitative comparison between different vesicles. A limited number of studies has dealt with this topic in detail in the past. Polar and charged molecules seem to have low permeability compared to nonpolar molecules,^44^ and an increased permeability for certain low molecular weight compounds has been observed for GUVs compared to LUVs^43^ as well as the co-existence of low and high permeability GUV populations.^44^ Here, we focused on the leakiness of immobilized GUVs and if immobilization strength can induce leakage. Our data shows that a negative impact of strong adhesion was only observed for GUVs prepared by PVA formation but not with Pt wire GUVs. Reasons for these observations are unclear and outside of the scope of this work. It has been shown that trace amounts of PVA in or on the membrane can alter membrane property as suggested by Dao *et al.*^60^ that could promote membrane defects. In addition, altered membrane properties such as the bending rigidity in the presence of salt might contribute,^76,77,81^ which could explain the increased leakage of Pt wire GUVs in absence of salt. Our measurements at low streptavidin concentrations show that careful control of vesicle adhesion allows for efficient immobilization of GUVs while not compromising the membrane integrity.

Proton permeability is difficult to quantify and results described in the literature vary greatly, likely owing to differences in experimental conditions.^82,83^ Here, proton permeability was tested using GUVs with encapsulated HPTS in the presence of a proton gradient. The results of the HPTS leakage experiments helped us to settle for immobilization conditions that showed immobilization and minimal HPTS leakage. The proton gradient was applied by exterior washing of the GUVs, thus mimicking standard procedures during a biochemical experiment. Only minimal increase in the HPTS ratio was observed during the presence of a proton gradient and most GUVs showed a response to the addition of the protonophore gramicidin, indicating a low level of proton leakage. Our data suggests that immobilization can and must be fine-tuned depending on the experimental set-up. The composition of the membrane and the presence of MP has also been reported to influence the proton permeabilty.^24,84,85^ Using a microfluidic approach, Dimova and colleagues found a slightly lower proton permeability for GUVs compared to LUVs.^24^ Encouragingly for studies of vectorial proton transport experiments, GUVs between 5 and 20 μm that retain HPTS seem to be also tight against protons.

Different strategies have been described to reconstitute membrane proteins into GUVs. Partial dehydration of deposited membrane protein before lipid rehydration seems to work well for some proteins, but a milder procedure might be suitable for large multisubunit complexes such as the members of the respiratory chain or eukaryotic proteins. Reconstitution of membrane proteins into SUVs is not straightforward and often was only established with considerable effort. To avoid similar efforts for the reconstitution into GUVs, protein transfer from SUVs to GUVs *via* membrane fusion is an attractive strategy. We and others have successfully applied charge mediated fusion for the insertion of SUV embedded proteins into the GUV membrane, retaining their functionality, and it is currently the method of choice in our lab and was also applied here.^46,47^ The effect of salt on the fusion of liposomes with oppositely charged lipids has not been conclusively discussed in the literature as some observed no or little influence^46,68^ and others reported impeded fusion in presence of salt.^47^ Using our protocols, we clearly observe rapid and efficient fusion of unlabeled GUVs with fluorescently labeled SUVs both in the presence and absence of salt. However, the fluorescence intensity observed in the GUV membrane is reduced to 50% if fusion is performed in presence of 100 mM NaCl, although the fusion kinetics are similar under both conditions. In fusion experiments with non-immobilized GUVs, PVA GUVs still show a slightly decreased fusion behavior in presence of salt, but the difference was almost gone in Pt wire GUVs. We speculate that this might result from different lipid composition of the GUV membrane, *i.e.* asymmetry of composition in the two leaflets after electroformation^86^ or that trace amounts of PVA might influence the fusion behavior in presence of salt.^60^

Finally, proteoliposomes containing labeled lipid and labeled *bo*_3_ oxidase or no protein were mixed in different ratios and subjected to fusion with unlabeled GUVs. For each of the mixtures, a linear dependence between *bo*_3_ oxidase signal and lipid-coupled dye was observed, indicating that the relative amount of protein can be correlated from the relative amount of lipid. In addition, the different slopes of the mixture indicate that empty and protein containing SUVs show similar fusion behaviour, that allows to control the protein content of GUVs with variation of the empty liposome content. This is useful in experiments, where different protein densities are required. The described method requires only a single protein reconstitution in SUVs for different protein content in GUVs thereby minimizing protein usage and experimental variation. Unfortunately, the method is not suited to distinguish between full fusion and hemifusion or simple adhesion of the vesicles to the GUV membrane, which is not trivial and not currently established as a convenient method. Our experiment allows estimation of relative protein incorporation in different vesicles of the same experiment without the need of protein labeling. This is an important information to correlate the observed transport signal to the number of proteins involved on a per vesicle basis and is an important step towards better quantification of GUV experiments. While we did not observe a GUV signal increase if neutral SUVs were used, positively charged SUVs might still be able to adhere to vesicles in the presence of salt and undergo lipid mixing without fusion, as suggested by Ishmukhametov *et al.*^47^ In our previous experiments, however, we were able to show that fusion of SUV and GUV yields functional GUVs and that the fusion behavior was similar to fusion events between oppositely charged SUVs.^46^ To unambiguously discriminate between content or lipid mixing, assays in which fusion of both membrane leaflets is a prerequisite to trigger a signal change are required. However, aside from enzyme-mediated assays, classical content mixing assays seem difficult due to unwanted interaction of the often negatively charged cargo with the positively charged lipids. Recently, Lira *et al.*^68^ described a FRET based assay allowing them to distinguish between hemifusion and full fusion, opening new avenues for verification of complete membrane fusion.

## Experimental

### Materials

1,2-Dioleoyl-*sn-glycero*-3-phosphocholine (DOPC), 1,2-dioleoyl-*sn-glycero*-3-phospho-(1′-rac-glycerol) (sodium salt) (DOPG) 1,2-dioleoyl-3-trimethylammonium-propane (chloride salt) (DOTAP), 1,2-distearoyl-*sn-glycero*-3-phosphoethanolamine-*N*-[biotinyl(polyethylene glycol)-2000] (ammonium salt) (DSPE-PEG(2000) Biotin) and 1,2-dioleoyl-*sn-glycero*-3-phosphoethanolamine-*N*-(lissamine rhodamine B sulfonyl) (ammonium salt) (Liss Rhod PE) were obtained from Avanti Polar Lipids (Alabaster, AL, USA), streptavidin from IBA-Lifesciences (Göttingen, Germany), Invitrogen™ 8-hydroxypyrene-1,3,6-trisulfonic acid, trisodium salt (HPTS) from Thermo Fisher Scientific (Waltham, Massachusetts, USA), DY-647P1 Maleimide from Dyomics (Jena, Germany) and Polyvinyl alcohol (PVA), fully hydrolyzed, molecular weight approximately 145 000 for synthesis from Merck (Darmstadt, Germany). Other chemicals were obtained from Sigma (St. Louis, Missouri, USA).

### PVA assisted GUV formation

GUV formation with PVA was done as described^50^ with a few modifications. 1 mL 5% PVA (w/v) in 200 mM sucrose was incubated for 1 h at 90 °C in a thermal shaker lite (VWR international GmbH, Dietikon, Switzerland) at 1000 rpm, vortexing every 15–20 min. A coverglass (25 mm Ø # 1.0, VWR international GmbH) was rinsed with 70% ethanol and placed on an aluminum-foil-covered heat plate set to 50 °C. Rubber O-rings with 20 mm diameter and 1.4 mm thickness were placed centrally on top of the coverglass. 200 μL PVA solution was pipetted onto the coverglass area inside the rubber ring and the gel was left to dry for 1 h at 50 °C. 20 μL of lipids dissolved in chloroform at 1 mg mL^−1^ composed of 68.8 mol% DOPC, 30 mol% DOPG, 1 mol% Liss Rhod PE and 0.2 mol% DSPE–PEG(2000) Biotin were evenly distributed onto the gels using a 10 μL syringe (MICROLITER^TM^ #701, Hamilton, Bonaduz, Switzerland). Solvent was evaporated for 1.5–2 h under vacuum, after which lipids were rehydrated for 1 h using 500 μL formation buffer (5 mM MOPS-BTP pH 7.4, 200 mM sucrose and salt as indicated). The solution was removed from the wells and GUVs were stored for at least 1 h at 4 °C before performing further experiments.

### Pt wire GUV electroformation

Electroformation of GUVs using platinum (Pt) wires was performed as described previously^58,87^ with minor modifications. Pt wires (electrode distance 2 mm) were cleaned by hand using soap before incubation in 94% EtOH for 10 min, followed by incubation in chloroform for 10 min, both performed in a sonication bath. Pt wires were dried at RT at 1 atm for 10 min before applying lipid solution to the wires. 10 μL of lipids dissolved in chloroform at 2 mg mL^−1^ with lipid mixture as described were evenly deposited on the wires using a 10 μL Hamilton syringe. Lipids were dried on the wires for 1 h in a desiccator under vacuum. For every chamber a coverglass (25 mm Ø) was coated in a 0.1 g mL^−1^ milk powder solution for 30 min at RT and mild agitation. The coverglass was rinsed with dH_2_O and glued to the formation chamber using Dublisil 22 plus silicone (Dreve Dentamid GmBH, Unna, Germany). Pt wires were connected to an AC electric field generator (PCGU1000, Velleman Group, Gavere, Belgium) using JST XH2.54 cable with 2 pin female socket (Play-Zone GmbH, Steinhausen, Switzerland) connected to a BNC male to 2 pin terminal block cable (Delock, Berlin, Germany). The formation chamber was filled with 800 μL formation buffer. The following wave sequence protocol was applied: 5 min at 0.44*V*_PP_, 5 min at 0.88*V*_PP_, 15 min at 1.32*V*_PP_, 30 min at 1.76*V*_PP_ and overnight at 2.2*V*_PP_. Every step was performed at 500 Hz.

### ITO GUV electroformation

Electroformation using indium tin oxide (ITO) coated glass slides was performed as described previously^58,87^ with minor modifications. ITO coated slides were cleaned by hand using soap and incubated in 94% EtOH for 10 min in a sonication bath. 10 μL of lipids dissolved in chloroform at 1 mg mL^−1^ with lipid mixture as described were evenly distributed on two separate slides on the conductive surface. The slides were dried for 1 h in a desiccator under vacuum. The electroformation chamber was assembled in a custom 3D printed slide holder (File S1, ESI†) using a 1.00 mm rubber spacer and filled with 600 μL formation buffer. The slides were connected to an AC electric field generator (PCGU1000, Velleman Group, Gavere, Belgium) using crocodile clamps (JYE BNC, Play-Zone GmbH, Steinhausen, Switzerland). The same wave sequence protocol was applied as for Pt wire formation.

### Imaging slide preparation

Imaging of GUVs was performed in an 8 well chambered glass slide (#1.5 high performance cover glass, Cellvis, Mountain View, California, USA). To immobilize GUVs, wells were coated using 200 μL T50-buffer (10 mM Tris–HCL pH 8, 50 mM NaCl) containing 50 μg mL^−1^ biotinylated BSA for 30 min at RT with mild agitation. The solution was replaced by 200 μL T50 buffer containing 10 μg mL^−1^ streptavidin unless stated otherwise and incubated as above. Wells were washed once using 200 μL imaging buffer (5 mM MOPS-BTP pH 7.4, 200 mM glucose and salt as indicated). A desired amount of GUVs (10–100 μL) was loaded into imaging buffer with a final volume of 400 μL and GUVs were left to settle for 1 h prior to imaging. For experiments where immobilization was not required, GUVs were imaged in wells coated using only 200 μL T50-buffer containing 50 μg mL^−1^ BSA for 30 min. Washing and GUV loading was performed as described.

### Image acquisition

Microscopy slides were imaged using an inverted fluorescence microscope (Nikon Ti-2 Eclipse with Crest X-light V2 spinning disk module (disk unit 60 μm), Nikon Europe BV, Amsterdam, Netherlands) with a CFI Plan Fluor 40× oil immersion objective (CFI Plan Fluor 40×/1.30 W.D. 0.24, Nikon Europe BV). Brightfield and fluorescence images were recorded by an Andor Zyla 4.2 Plus USB3 camera in Widefield and Spinning Disk Confocal mode using LED light excitation. HPTS was imaged with excitation at 395 nm and 470 nm and emission at 515 nm using appropriate exciter, emitter, dichroic filter cubes. Liss Rhod PE was imaged with excitation at 550 nm and emission at 595 nm and DY-647P1 with excitation at 640 nm and emission at 698 nm using appropriate exciter, emitter, dichroic filter cubes. Z-Stacks were recorded in confocal mode from top to bottom (below the slide surface) with a step size of 1 μm and 72 steps. Time series were recorded as indicated.

### Automatic detection of GUVs

Images in the Liss Rhod PE channel were analyzed in FIJI.^88^ For Z-stack acquisitions, two custom macros were used to extract the data. With the first macro a background subtraction is performed on all slices of the Z-stack with a rolling ball radius of 100.0, then an average projection of the stack is made and a bandpass filter is applied to improve separation of nearby vesicles by highlighting the contrast between the background and vesicles using the following settings: filter large set to 40 pixels, filter small set to 5 pixels, suppress stripes set to None, tolerance of direction set to 5%, with autoscale after filtering and saturation of image when autoscaling enabled. Manual thresholding is performed to create a binary mask separating vesicles and background. The second macro cleans up the mask by performing the binary processes Fill Holes, Erode and Watershed and vesicles are identified using the Analyze Particles function with Size set to 0.75 μm^2^ – Infinity, Circularity set to 0.70–1.00 and Display Results, Exclude on Edges and Add to ROI manager enabled. For single images, the use of the first macro was omitted and manual thresholding was directly performed on the image. The second macro was then used as described, with Size set to 19.6–314.1 μm^2^ (corresponding to vesicles with diameters between 5 and 20 μm).

### Characterization of GUV formation

For characterization of GUV formations, GUVs were formed as described and 10 μL PVA GUVs or 100 μL electroformed GUVs were loaded to imaging wells coated with BSA as described. 9 Z-Stacks were recorded in each well using a 3 × 3 multi spot acquisition from the top left corner of a well to the bottom right corner. GUVs were automatically identified from the *Z* stack as described. Using the Measure function in FIJI, the Feret diameter of the identified particles is obtained. To estimate the concentration of GUVs with a diameter between 5–20 μm, the number of identified particles with the according Feret diameters were counted. Based on the image dimensions (332.8 μm × 332.8 μm), a total area of 0.9968 mm^2^ for all 9 images was obtained which was used to calculate the amount of GUVs per mm^2^. This value was then multiplied by the area of the well which was taken as 80.91 mm^2^, according to the manufacturer, to estimate the amount of GUVs in the entire well chamber. This number was then divided by the volume of GUV solution which was added to the well to obtain the concentration of GUVs (5–20 μm) in solution. As a measure for the quality of the formation to produce vesicles with the desired size, the number of particles (5–20 μm) was divided by the total number of particles detected as described.

### Calculation of Lipid Yield

To estimate the lipid yield, the total surface area *A*_GUV_ of each vesicle was calculated using eqn (1) based on the measured Feret diameter *d*_GUV_ and the bilayer thickness *d*_bilayer_ which was assumed to be 5 nm.1
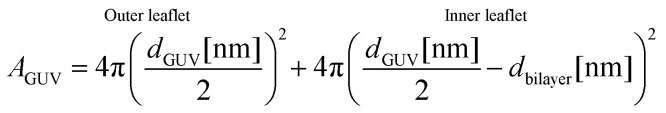
The lipid yield was calculated using eqn (2)2
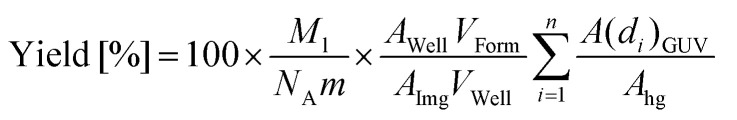
The total number of lipids in GUV *i* with a diameter of *d*_*i*_ was calculated by dividing the surface *A*(*d*_*i*_)_GUV_ calculated according to eqn (1) by the surface area of the lipid headgroup *A*_hg_ which was assumed to be 0.71 nm^2^.^89,90^ The total number of lipids from all GUVs was calculated by summation of the lipid number of each GUV *i* with *n* as the total number of particles detected in one formation condition. To estimate the total number of lipids in the formation solution after removal from the formation chamber, the sum was divided by the total imaged area *A*_Img_ of the 9 images (0.9968 mm^2^) times the volume *V*_Well_ of GUV solution added to the well (for example 10 μL for PVA GUVs) and multiplied with the total well area *A*_Well_ (80.91 mm^2^) times the volume *V*_Form_ of solution used for the formation of GUVs (for example 500 μL for PVA GUVs). The yield was then obtained by dividing the total number of lipids in the formation solution by the Avogadro constant *N*_A_, multiplying with the average molecular weight *M*_l_ of the lipids which was assumed to be 790 g mol^−1^, based on a lipid composition of roughly 70 mol% DOPC and 30 mol% DOPG, and finally divided by the lipid weight *m* used to coat the surface for GUV formation (20 μg). This was multiplied by 100 to obtain values in percentage.

### Immobilization assay

Flow immobilization experiments were performed in channel slides (μ-Slide III 3D Perfusion uncoated, ibidi GmbH, Gräfelfing, Germany) connected to a syringe pump (Ossila, Sheffield, UK). All experiments were performed at a flow rate of 5 μL s^−1^. The slide was coated as described using 30 μL 100 μg mL^−1^ biotinylated BSA followed by 30 μL 0, 10 or 100 μg mL^−1^ streptavidin in T50 buffer. 1 μL 0 mM NaCl and 5 μL 100 mM NaCl PVA GUVs were loaded into imaging buffer to a final volume of 30 μL and GUVs were left to settle for 1 h. Prior to imaging the slide was sealed and the channels were filled with imaging buffer according to the manufacturer protocol. Image acquisition was performed using the confocal Liss Rhod PE settings and a time series was recorded over 2 min with a 0.5 s interval and 100 ms exposure at 50% laser intensity. Flow was started after 15 s using the syringe pump. GUVs were automatically detected as described on the last image before visible flow. To detect non-immobilized vesicles, all images recorded during flow were transformed into an average Z-projection, resulting in smearing and a decreased intensity for non-immobilized GUVs. This allowed automatic detection of immobilized GUVs by thresholding as described. The percentage of immobilized GUVs was calculated as the ratio between the detected number of GUVs on the average projection and before flow.

### Calculation of the streptavidin density

To achieve equal slide coating, the streptavidin amount per surface was calculated based on the microscopy well dimensions indicated by the slide manufacturers. For simplicity reasons, we will refer to this value as the streptavidin density. For 8 well chambered slides, well dimensions are 8.7 × 9.3 mm leading to a bottom surface of 80.91 mm^2^. Based on the coating volume of 200 μL, the height of the coated wall was estimated to be 2.47 mm, giving a total wall surface of 88.92 mm^2^ leading to approximately 169.83 mm^2^ of surface that is coated. This gives a volume to surface ratio of 1.18 μL mm^−2^. For ibidi slides, the well diameter is 5.5 mm with a well height including the channels of 1.7 mm. This gives a bottom surface of 23.75 mm^2^ and a wall surface of 29.37 mm^2^. The well is connected by two channels which have a width of 1 mm and a height of 0.5 mm, which takes approximately 1 mm^2^ away from the wall surface. This gives a total surface of 52.12 mm^2^ and a volume to surface ratio of 0.58 μL mm^−2^ with 30 μL coating volume which is approximately half of the ratio for the 8 well chambered slide. Thus, to get similar immobilization conditions for both slides, the concentration of streptavidin for the ibidi slides should be twice as high as for the 8 well chambered slide.

### HPTS leakage

For PVA GUVs, HPTS leakage during immobilization was measured at streptavidin densities of 0, 6, 30 and 60 ng mm^−2^ streptavidin as calculated above. HPTS was either added to imaging buffer before GUV addition or after immobilization. Final HPTS concentrations in the well chamber were 43–50 μM. 8 well chambered slides were prepared as described and 390 μL or 350 μL appropriate imaging buffer containing HPTS and 10 μL or 50 μL 0 mM NaCl or 100 mM NaCl GUVs were loaded, respectively. For leakage after immobilization, 375 μL appropriate imaging buffer as well as 25 μL GUV solution was loaded, whereby 5 μL 0 mM NaCl GUVs were diluted with 20 μL formation buffer and 100 mM NaCl were used undiluted. GUVs were left to settle, after which 100 μL appropriate imaging buffer containing 230 μM HPTS was added and incubated for 1 h before imaging. For Pt wire GUVs, HPTS leakage during immobilization was measured at coating densities of 6 and 60 ng mm^−2^ streptavidin. 8 well chambered slides were prepared as described and 380 μL or 350 μL appropriate imaging buffer containing HPTS and 20 μL or 50 μL 0 mM NaCl or 100 mM NaCl GUVs were loaded, respectively. GUVs were imaged by recording Z-stacks using the Liss Rhod PE channel and the 470 nm excitation channel for HPTS.

### HPTS leakage analysis

GUVs were automatically detected from Z-stacks as described. The number of GUVs considered for analysis was limited to 148 for each sample. 20 Background regions of interest (bgROIs) were drawn in by hand in areas that did not contain GUVs. For leakage analysis, a background subtraction with a rolling ball radius of 200 pixels and light background enabled was first performed on each slice of the Z-stack in the HPTS channel. An intensity profile of the GUVs and bgROIs in the *Z* dimension was recorded using the Time Series Analyzer V3 plugin in FIJI with the average intensity setting. The profiles were normalized to the average of the first 20 steps of the Z-stack. The profiles of the bgROIs were averaged to create a mean background profile. This was then subtracted from each GUV profile which were then inverted by multiplying with −1 to obtain positive values for GUVs that did not leak. The maximum intensity of the profile was then measured, indicating the largest absence of HPTS signal for each GUV. Only GUVs in the desired size range (5–20 μm diameter) were used for analysis. Because small GUVs tended to move at low streptavidin concentrations and were only imaged by very few slices, vesicles with diameters below 7 μm were further discarded as well. We further limited the number of vesicles from each experiment to 85 due to limitations in GraphPad Prism 8.0 which resulted in 20–85 vesicles that were analyzed. The median of the maximum intensity distribution of the GUVs was calculated in Prism and used to define a threshold for leakage based on the average median at low streptavidin concentrations. Leaky GUVs were defined by having a maximum intensity below approximately 25% of the average median. The percentage of leaky GUVs was calculated by dividing the number of GUVs below the threshold by the total number of GUVs.

### Proton leakage

For proton leakage, formation buffers contained 10 mM MOPS-KOH pH 7.4, 200 mM sucrose and imaging buffers were 10 mM MOPS-KOH pH 7.4, 200 mM glucose and 10 mM MOPS-KOH pH 8.0, 200 mM glucose. Buffers for GUVs with salt contained 100 mM NaCl as well. 8 well chambered slides for immobilization were prepared as descried. For PVA GUVs, 400 μL imaging buffer and 20 μL 0 mM NaCl or 50 μL 100 mM NaCl GUVs were loaded, respectively. For Pt wire GUVs, 400 μL imaging buffer and 50 μL GUVs were loaded, both for 0 mM NaCl GUVs and 100 mM NaCl GUVs. Before imaging, a pH exchange in the exterior solution was performed by performing two 1 mL wash steps with imaging buffer at pH 7.4 followed by two 1 mL wash steps with buffer at pH 8.0. Washing was performed using two 1 mL pipettes with simultaneous addition and removal of solution in a well chamber. A single image was acquired in confocal mode using the Liss Rhod PE channel and GUVs were then recorded in confocal mode using both HPTS channels for 60 min at 5 min intervals. Approximately 1 min after the 60 min acquisition (61 min), another time series was recorded for 5 min and 5 s intervals with addition of 5 μL 1 mM gramicidin (final concentration 12.5 μM) after 1 min (62 min). The total acquisition time was 66 min.

### Proton leakage analysis

GUVs were identified using the single Liss Rhod PE image as described. The HPTS intensity for both channels were extracted using the Time Series Analyzer V3 plugin in FIJI with the average intensity setting. The HPTS ratio was calculated by dividing the intensity at 470 nm excitation by the intensity at 395 nm excitation. To assess the amount of leakage after 20 min, the ratio at 0 min was subtracted from the ratio at 20 min. As we expect an increase in pH on the inside as GUVs leak, only vesicles with a ratio difference >0 were considered for further analysis. The ratio at 66 min was then subtracted from the ratio at 0 min, indicating the total possible increase for a GUV. The ratio difference 20–0 min was then divided by the total possible increase and multiplied with 100 to obtain a percentage of leakage.

### Preparation of SUVs

Liposomes were formed with a lipid composition of 69 mol% DOPC, 30 mol% DOTAP and 1 mol% Liss Rhod PE or 99 mol% DOPC and 1 mol% Liss Rhod PE. Lipids dissolved in chloroform were mixed in a 25 mL round bottom flask and chloroform was evaporated under a constant stream of N_2_ while rotating the flask. The film was further dried overnight in a desiccator under vacuum. Lipids were resuspended in formation buffer to a concentration of 5 mg mL^−1^ and unilamellar vesicles were obtained by performing seven freeze-thaw cycles and stored at −80 °C before further use. For fusion experiments, liposomes were thawed and diluted to 50 μg mL^−1^ using imaging buffer and size was adjusted by sonication on ice using a tip sonicator (Vibra Cell 75186, Thermo Fisher Scientific, Waltham USA) for 2 min with 30 s ON and 30 s OFF pulses and 40% amplitude.

### Cytochrome *bo*_3_ ubiquinol oxidase purification and labeling

Cytochrome *bo*_3_ ubiquinol oxidase mutant IIIA21C, encoded by a cysless pETcyoII plasmid, was expressed and purified as described.^91–93^ The protein was cysteine-labeled with DY-647P1 maleimide as previously published^94^ with minor modifications. The excess dye was removed by performing a CentriPure P50 (emp BIOTECH, Berlin, Deutschland) size exclusion chromatography followed by a Superdex 200 Increase 10/300 GL column (ÄKTA Pure system, GE Healthcare, Boston, Massachusetts, USA) purification at 4 °C. The labeled protein was concentrated to 20 μM.

### Reconstitution of cytochrome *bo*_3_ ubiquinol oxidase

DOTAP liposomes were formed and sonicated at 5 mg mL^−1^ as described. 240 μL liposomes were destabilized using a final concentration of 0.4% (w/v) sodium cholate, mixed with 12 μL purified *bo*_3_ oxidase (20 μM) and incubated on ice for 30 min to obtain proteoliposomes with a lipid-to-protein ratio of 40 (w/w). Empty liposomes were prepared by addition of 12 μL buffer instead of protein. Liposomes were collected by gel filtration using a CentriPure P10 column (*emp* BIOTECH GmbH, Berlin, Germany) pre-equilibrated with formation buffer. The column was eluted using 1.2 mL formation buffer to obtain 1 mg mL^−1^ liposomes. Prior to measuring, the liposomes were diluted to 0.2 mg mL^−1^ using formation buffer and proteoliposomes and empty liposomes were mixed in a 3/0, 2/1, 1/2 and 0/3 v/v ratio.

### Charge mediated fusion

Charge mediated fusion of negatively charged GUVs and positively charged SUVs was performed in 8 well chambered slides and in 1.5 mL Eppendorf tubes. GUVs were prepared as described and 10–50 μL GUV solution was diluted in formation buffer to a final volume of 50 μL to achieve similar vesicle concentrations. For fusion in an 8 well chambered slide, diluted GUVs were loaded into imaging buffer containing salt as indicated to a final volume of 400 μL and immobilized for 1 h. A time series was recorded for 5 min at 5 s intervals using confocal Liss Rhod PE settings for all experiments and additional DY-647P1 settings for experiments with proteoliposomes. Fusion was initiated by addition of 100 μL SUVs after 30 s. Final concentration of SUVs was 10 μg mL^−1^ for experiments with PVA and Pt wire GUVs and empty vesicles and 40 μg mL^−1^ for experiments with PVA GUVs and mixtures of empty and proteoliposomes. For fusion in Eppendorf tubes, diluted GUVs were mixed with 50 μL empty SUVs (20 μg mL^−1^) and incubated for 15 min at RT. 100 μL of fused GUVs were transferred into 400 μL imaging buffer and GUVs were immobilized for 1 h. Z-Stacks were recorded as described using confocal Liss Rhod PE settings.

### Analysis of charge mediated fusion

For fusion of PVA and Pt wire GUVs with empty SUVs in 8 well chambered slides, 10–50 GUVs were detected as described using a single Liss Rhod PE image recorded after 300 s. A bgROI was manually drawn next to every vesicle. The Liss Rhod PE intensity was extracted for every GUV and corresponding bgROI using the Time Series Analyzer V3 plugin in FIJI with the average intensity setting. For each GUV, the background was removed by subtracting the intensity of the corresponding bgROI. The mean intensity increase of GUVs per experiment was calculated and average and standard deviation of mean intensity values from four replicates were plotted. To show end point signal distributions, the final Liss Rhod PE intensity of individual GUVs of the four replicates were combined and plotted. For experiments using proteoliposomes, 30–50 GUVs were detected as described after 180 s. On the same image, mean grey intensity for Liss Rhod PE and DY-647P1 of GUVs and corresponding bgROIs were extracted using the Measure function of FIJI. Background was subtracted as described. To show signal correlation, the Liss Rhod PE signal of every GUV was plotted on the *X*-axis against the DY-647P1 signal of every GUV on the *Y*-axis. For fusion experiments in Eppendorf tubes, GUVs were automatically identified from Z-stacks as described. An average Z-projection was performed and the background was subtracted using a rolling ball radius of 200 pixels. Using the Measure function in FIJI, the mean grey intensity of the Liss Rhod PE channel was extracted for every GUV and plotted as a signal distribution.

## Conclusions

The application of GUVs in a variety of biochemical and biophysical disciplines is very attractive and a broad variety of fascinating results have been described. In this work, we want to contribute to a broader application of GUVs as a system of choice for the investigation of membrane proteins incorporated into the GUV membrane, elevating GUVs from their current main role as sealed lipid container or membrane mimicking system used for docking or membrane deformation studies. We therefore have somewhat systematically analyzed existing methods for GUV formation and immobilization that are accessible without specialized equipment or knowledge and tested relevant properties for membrane protein reconstitution and substrate transport. Although both PVA assisted and electroformation methods used here are based on solvent-free systems, they differ in some of the investigated properties. Electroformation has been the method of choice for many researchers as it is well established and produces clean GUVs. We find that polymer assisted swelling has several advantages, like its ease of use, versatility, scalability, and the production of high GUV numbers, but further research in other materials than PVA and agarose is needed to establish whether polymer assisted swelling can produce GUVs free of impurities.^60,95^ Obviously, powerful alternatives not described here exist, such as microfluidic or oil-emulsion techniques for GUV generation or detergent-mediated reconstitution techniques. There are also efforts to replace natural lipids as used here by more robust synthetic polymers,^24,85,96–98^ and while they surpass GUV properties in terms of robustness towards mechanical and chemical stresses and life span, they are rather unlikely to match the properties of natural lipids to accommodate for membrane protein thickness and annular lipid layer. GUVs are thus designated to play a critical role also in the future, and robust and widely applicable methods for their generation and use is of importance for all researchers interested.

## Conflicts of interest

There are no conflicts to declare.

## Supplementary Material

SM-018-D2SM00551D-s001

SM-018-D2SM00551D-s002

SM-018-D2SM00551D-s003

## References

[cit1] Dimova R. and Marques C., in The Giant Vesicle Book. ed. R. Dimova and C. Marques, CRC Press, Boca Raton, FL, USA, 1st edn, 2019, vol. 53, p. 676

[cit2] Biner O., Schick T., Ganguin A. A., von Ballmoos C. (2018). Towards a Synthetic Mitochondrion. Chim. Int. J. Chem..

[cit3] Amati A. M., Graf S., Deutschmann S., Dolder N., von Ballmoos C. (2020). Current problems and future avenues in proteoliposome research. Biochem. Soc. Trans..

[cit4] Wang X., Du H., Wang Z., Mu W., Han X. (2021). Versatile Phospholipid Assemblies for Functional Synthetic Cells and Artificial Tissues. Adv. Mater..

[cit5] Staufer O., Antona S., Zhang D., Csatári J., Schröter M., Janiesch J. W. (2021). *et al.*, Microfluidic production and characterization of biofunctionalized giant unilamellar vesicles for targeted intracellular cargo delivery. Biomaterials.

[cit6] Luckey M. , Membrane Structural Biology: With Biochemical and Biophysical Foundations. Cambridge: Cambridge University Press, 2008. Available from: https://ebooks.cambridge.org/ref/id/CBO9780511811098

[cit7] Kobliakov V. A. (2017). Role of proton pumps in tumorigenesis. Biochem..

[cit8] Licon-Munoz Y., Fordyce C. A., Hayek S. R., Parra K. J. (2018). V-ATPase-dependent repression of androgen receptor in prostate cancer cells. Oncotarget.

[cit9] Hwang S. M., Lee J. Y., Park C. K., Kim Y. H. (2021). The Role of TRP Channels and PMCA in Brain Disorders: Intracellular Calcium and pH Homeostasis. Front. Cell Dev. Biol..

[cit10] Deisl C., Albano G., Fuster D. G. (2014). Role of Na/H exchange in insulin secretion by islet cells. Curr. Opin. Nephrol. Hypertens..

[cit11] Bull S. C., Doig A. J. (2015). Properties of protein drug target classes. PLoS One.

[cit12] Von Ballmoos C., Biner O., Nilsson T., Brzezinski P. (2016). Mimicking respiratory phosphorylation using purified enzymes. Biochim. Biophys. Acta, Bioenerg..

[cit13] Lundgren C. A. K., Sjöstrand D., Biner O., Bennett M., Rudling A., Johansson A. (2018). *et al.*, Scavenging of superoxide by a membrane-bound superoxide oxidase. Nat. Chem. Biol..

[cit14] Graf S. S., Hong S., Müller P., Gennis R., von Ballmoos C. (2021). Energy transfer between the nicotinamide nucleotide transhydrogenase and ATP synthase of *Escherichia coli*. Sci. Rep..

[cit15] Kahya N., Pécheur E. I., De Boeij W. P., Wiersma D. A., Hoekstra D. (2001). Reconstitution of membrane proteins into giant unilamellar vesicles via peptide-induced fusion. Biophys. J..

[cit16] Girard P., Pécréaux J., Lenoir G., Falson P., Rigaud J. L., Bassereau P. (2004). A new method for the reconstitution of membrane proteins into giant unilamellar vesicles. Biophys. J..

[cit17] Dezi M., Di Cicco A., Bassereau P., Levy D. (2013). Detergent-mediated incorporation of transmembrane proteins in giant unilamellar vesicles with controlled physiological contents. Proc. Natl. Acad. Sci. U. S. A..

[cit18] Hansen J. S., Elbing K., Thompson J. R., Malmstadt N., Lindkvist-Petersson K. (2015). Glucose transport machinery reconstituted in cell models. Chem. Commun..

[cit19] Bian T., Autry J. M., Casemore D., Li J., Thomas D. D., He G. (2016). *et al.*, Direct detection of SERCA calcium transport and small-molecule inhibition in giant unilamellar vesicles. Biochem. Biophys. Res. Commun..

[cit20] Altamura E., Milano F., Tangorra R. R., Trotta M., Omar O. H., Stano P. (2017). *et al.*, Highly oriented photosynthetic reaction centers generate a proton gradient in synthetic protocells. Proc. Natl. Acad. Sci. U. S. A..

[cit21] Almendro-Vedia V. G., Natale P., Mell M., Bonneau S., Monroy F., Joubert F. (2017). *et al.*, Nonequilibrium fluctuations of lipid membranes by the rotating motor protein F_1_F_0_-ATP synthase. Proc. Natl. Acad. Sci. U. S. A..

[cit22] Lee K. Y., Park S.-J., Lee K. A., Kim S.-H., Kim H., Meroz Y. (2018). *et al.*, Photosynthetic artificial organelles sustain and control ATP-dependent reactions in a protocellular system. Nat. Biotechnol..

[cit23] Han W. B., Kang D. H., Na J. H., Yu Y. G., Kim T. S. (2019). Enhancement of membrane protein reconstitution on 3D free-standing lipid bilayer array in a microfluidic channel. Biosens. Bioelectron..

[cit24] Marušič N., Otrin L., Zhao Z., Lira R. B., Kyrilis F. L., Hamdi F. (2020). *et al.*, Constructing artificial respiratory chain in polymer compartments: Insights into the interplay between *bo*_3_ oxidase and the membrane. Proc. Natl. Acad. Sci. U. S. A..

[cit25] Diederichs T., Tampé R. (2021). Single Cell-like Systems Reveal Active Unidirectional and Light-Controlled Transport by Nanomachineries. ACS Nano.

[cit26] Parker J. L., Mindell J. A., Newstead S. (2014). Thermodynamic evidence for a dual transport mechanism in a POT peptide transporter. eLife.

[cit27] Walde P., Cosentino K., Engel H., Stano P. (2010). Giant vesicles: preparations and applications. ChemBioChem.

[cit28] Rideau E. , Wurm F. R. and Landfester K., Self-Assembly of Giant Unilamellar Vesicles by Film Hydration Methodologies, in Advanced Biosystems. John Wiley & Sons, Ltd, 2019, p. 1800324 10.1002/adbi.201800324 32648708

[cit29] Yuan W., Piao J., Dong Y. (2021). Advancements in the preparation methods of artificial cell membranes with lipids. Mater. Chem. Front..

[cit30] Robinson T. , Microfluidics and giant vesicles: creation, capture, and applications for biomembranes, in Advances in Biomembranes and Lipid Self-Assembly, Elsevier Ltd, 2019, pp. 271–315 10.1016/bs.abl.2019.10.003

[cit31] Reccius C. H., Fromherz P. (2004). Giant Lipid Vesicles Impaled with Glass Microelectrodes: GigaOhm Seal by Membrane Spreading. Langmuir.

[cit32] Robinson T., Kuhn P., Eyer K., Dittrich P. S. (2013). Microfluidic trapping of giant unilamellar vesicles to study transport through a membrane pore. Biomicrofluidics.

[cit33] Yandrapalli N., Robinson T. (2019). Ultra-high capacity microfluidic trapping of giant vesicles for high-throughput membrane studies. Lab Chip.

[cit34] Robinson T. (2019). Microfluidic Handling and Analysis of Giant Vesicles for Use as Artificial Cells: A Review. Adv. Biosyst..

[cit35] Lira R. B., Steinkühler J., Knorr R. L., Dimova R., Riske K. A. (2016). Posing for a picture: Vesicle immobilization in agarose gel. Sci. Rep..

[cit36] Christensen S. M., Stamou D. (2007). Surface-based lipid vesicle reactor systems: Fabrication and applications. Soft Matter.

[cit37] Ritz S., Eisele K., Dorn J., Ding S., Vollmer D., Pütz S. (2010). *et al.*, Cationized albumin-biocoatings for the immobilization of lipid vesicles. Biointerphases.

[cit38] Sarmento M. J., Prieto M., Fernandes F. (2012). Reorganization of lipid domain distribution in giant unilamellar vesicles upon immobilization with different membrane tethers. Biochim. Biophys. Acta, Biomembr..

[cit39] Jain A., Cheng K. (2017). The principles and applications of avidin-based nanoparticles in drug delivery and diagnosis. J. Controlled Release.

[cit40] Bernard A. L., Guedeau-Boudeville M. A., Jullien L., Di Meglio J. M. (2000). Strong adhesion of giant vesicles on surfaces: dynamics and permeability. Langmuir.

[cit41] Zhdanov V. P., Kasemo B. (2002). Comments on rupture of adsorbed vesicles. Langmuir.

[cit42] Karatekin E., Sandre O., Guitouni H., Borghi N., Puech P. H., Brochard-Wyart F. (2003). Cascades of transient pores in giant vesicles: line tension and transport. Biophys. J..

[cit43] Fischer A., Oberholzer T., Luisi P. L. (2000). Giant vesicles as models to study the interactions between membranes and proteins. Biochim. Biophys. Acta, Biomembr..

[cit44] Nishimura K. K., Matsuura T., Sunami T., Fujii S., Nishimura K. K., Suzuki H. (2014). *et al.*, Identification of giant unilamellar vesicles with permeability to small charged molecules. RSC Adv..

[cit45] Jorgensen I. L., Kemmer G. C., Pomorski T. G. (2017). Membrane protein reconstitution into giant unilamellar vesicles: a review on current techniques. Eur. Biophys. J..

[cit46] Biner O., Schick T., Müller Y., von Ballmoos C. (2016). Delivery of membrane proteins into small and giant unilamellar vesicles by charge-mediated fusion. FEBS Lett..

[cit47] Ishmukhametov R. R., Russell A. N., Berry R. M. (2016). A modular platform for one-step assembly of multi-component membrane systems by fusion of charged proteoliposomes. Nat. Commun..

[cit48] Galkin M. A., Russell A. N., Vik S. B., Berry R. M., Ishmukhametov R. R. (2018). Detergent-free ultrafast reconstitution of membrane proteins into lipid bilayers using fusogenic complementary-charged proteoliposomes. J. Vis. Exp..

[cit49] Horger K. S., Estes D. J., Capone R., Mayer M. (2009). Films of Agarose Enable Rapid Formation of Giant Liposomes in Solutions of Physiologic Ionic Strength. J. Am. Chem. Soc..

[cit50] Weinberger A., Tsai F.-C., Koenderink G. H., Schmidt T. F., Itri R., Meier W. (2013). *et al.*, Gel-assisted formation of giant unilamellar vesicles. Biophys. J..

[cit51] Stein H., Spindler S., Bonakdar N., Wang C., Sandoghdar V. (2017). Production of Isolated Giant Unilamellar Vesicles under High Salt Concentrations. Front. Physiol..

[cit52] Parigoris E., Dunkelmann D. L., Murphy A., Wili N., Kaech A., Dumrese C. (2020). *et al.*, Facile generation of giant unilamellar vesicles using polyacrylamide gels. Sci. Rep..

[cit53] Angelova M. I., Dimitrov D. S. (1986). Liposome electroformation. Faraday Discuss Chem. Soc..

[cit54] López Mora N., Hansen J. S., Gao Y., Ronald A. A., Kieltyka R., Malmstadt N. (2014). *et al.*, Preparation of size tunable giant vesicles from cross-linked dextran(ethylene glycol) hydrogels. Chem. Commun..

[cit55] Girish V., Pazzi J., Li A., Subramaniam A. B. (2019). Fabrics of Diverse Chemistries Promote the Formation of Giant Vesicles from Phospholipids and Amphiphilic Block Copolymers. Langmuir.

[cit56] Pott T., Bouvrais H., Méléard P. (2008). Giant unilamellar vesicle formation under physiologically relevant conditions. Chem. Phys. Lipids.

[cit57] Li Q., Wang X., Ma S., Zhang Y., Han X. (2016). Electroformation of giant unilamellar vesicles in saline solution. Colloids Surf., B.

[cit58] Lefrançois P., Goudeau B., Arbault S. (2018). Electroformation of phospholipid giant unilamellar vesicles in physiological phosphate buffer. Int. Biol..

[cit59] Herold C., Chwastek G., Schwille P., Petrov E. P. (2012). Efficient Electroformation of Supergiant Unilamellar Vesicles Containing Cationic Lipids on ITO-Coated Electrodes. Langmuir.

[cit60] Dao T. P. T., Fauquignon M., Fernandes F., Ibarboure E., Vax A., Prieto M. (2017). *et al.*, Membrane properties of giant polymer and lipid vesicles obtained by electroformation and pva gel-assisted hydration methods. Colloids Surf., A.

[cit61] Souissi M., Pernier J., Rossier O., Giannone G., Le Clainche C., Helfer E. (2021). *et al.*, Integrin-functionalised giant unilamellar vesicles via gel-assisted formation: Good practices and pitfalls. Int. J. Mol. Sci..

[cit62] Stamou D., Duschl C., Delamarche E., Vogel H. (2003). Self-Assembled Microarrays of Attoliter Molecular Vessels. Angew. Chemie.

[cit63] Pick H., Alves A. C., Vogel H. (2018). Single-Vesicle Assays Using Liposomes and Cell-Derived Vesicles: From Modeling Complex Membrane Processes to Synthetic Biology and Biomedical Applications. Chem. Rev..

[cit64] Clement N. R., Gould J. M. (1981). Pyranine (8-hydroxy-1,3,6-pyrenetrisulfonate) as a probe of internal aqueous hydrogen ion concentration in phospholipid vesicles. Biochemistry.

[cit65] Han J., Burgess K. (2010). Fluorescent indicators for intracellular pH. Chem. Rev..

[cit66] Avnir Y., Barenholz Y. (2005). pH determination by pyranine: Medium-related artifacts and their correction. Anal. Biochem..

[cit67] Makino K., Shibata A. (2006). Surface Properties of Liposomes Depending on Their Composition. Adv. Planar Lipid Bilayers Liposomes.

[cit68] Lira R. B., Robinson T., Dimova R., Riske K. A. (2019). Highly Efficient Protein-free Membrane Fusion: A Giant Vesicle Study. Biophys. J..

[cit69] Garten M., Aimon S., Bassereau P., Toombes G. E. S. (2015). Reconstitution of a transmembrane protein, the voltage-gated ion channel, KvAP, into giant unilamellar vesicles for microscopy and patch clamp studies. J. Vis. Exp..

[cit70] Pazzi J., Subramaniam A. B. (2020). Nanoscale Curvature Promotes High Yield Spontaneous Formation of Cell-Mimetic Giant Vesicles on Nanocellulose Paper. ACS Appl. Mater. Interfaces.

[cit71] Peruzzi J., Gutierrez M. G., Mansfield K., Malmstadt N. (2016). Dynamics of Hydrogel-Assisted Giant Unilamellar Vesicle Formation from Unsaturated Lipid Systems. Langmuir.

[cit72] Movsesian N., Tittensor M., Dianat G., Gupta M., Malmstadt N. (2018). Giant Lipid Vesicle Formation Using Vapor-Deposited Charged Porous Polymers. Langmuir.

[cit73] Gleisner M., Kroppen B., Fricke C., Teske N., Kliesch T.-T., Janshoff A. (2016). *et al.*, Epsin N-terminal Homology Domain (ENTH) Activity as a Function of Membrane Tension. J. Biol. Chem..

[cit74] Seantier B., Kasemo B. (2009). Influence of Mono- And Divalent Ions on the Formation of Supported Phospholipid Bilayers via Vesicle Adsorption. Langmuir.

[cit75] Bouvrais H., Duelund L., Ipsen J. H. (2014). Buffers affect the bending rigidity of model lipid membranes. Langmuir.

[cit76] Faizi H. A., Frey S. L., Steinkühler J., Dimova R., Vlahovska P. M. (2019). Bending rigidity of charged lipid bilayer membranes. Soft Matter.

[cit77] De Mel J. U., Gupta S., Perera R. M., Ngo L., Zolnierczuk P., Bleuel M. (2020). *et al.*, Influence of External NaCl Salt on Membrane Rigidity of Neutral DOPC Vesicles. Langmuir.

[cit78] Holmberg A., Blomstergren A., Nord O., Lukacs M., Lundeberg J., Uhlén M. (2005). The biotin-streptavidin interaction can be reversibly broken using water at elevated temperatures. Electrophoresis.

[cit79] Weiru D., Wright L. D. (1964). Heat Stability of Avidin and Avidin-Biotin Complex and Influence of Ionic Strength on Affinity of Avidin for Biotin. Proc. Soc. Exp. Biol. Med..

[cit80] Kliesch T.-T., Dietz J., Turco L., Halder P., Polo E., Tarantola M. (2017). *et al.*, Membrane tension increases fusion efficiency of model membranes in the presence of SNAREs. Sci. Rep..

[cit81] Hac A., Heimburg T., Grubmu H., Bo R. A. (2003). Effect of Sodium Chloride on a Lipid Bilayer. Biophys. J..

[cit82] Deamer D. W., Nichols J. W. (1983). Proton-hydroxide permeability of liposomes. Proc. Natl. Acad. Sci. U. S. A..

[cit83] Paula S., Volkov A. G., Van Hoek A. N., Haines T. H., Deamer D. W. (1996). Permeation of protons, potassium ions, and small polar molecules through phospholipid bilayers as a function of membrane thickness. Biophys. J..

[cit84] Tsai M. F., Miller C. (2013). Substrate selectivity in arginine-dependent acid resistance in enteric bacteria. Proc. Natl. Acad. Sci. U. S. A..

[cit85] Kleineberg C., Wölfer C., Abbasnia A., Pischel D., Bednarz C., Ivanov I. (2020). *et al.*, Light-Driven ATP Regeneration in Diblock/Grafted Hybrid Vesicles. ChemBioChem.

[cit86] Steinkühler J., De Tillieux P., Knorr R. L., Lipowsky R., Dimova R. (2018). Charged giant unilamellar vesicles prepared by electroformation exhibit nanotubes and transbilayer lipid asymmetry. Sci. Rep..

[cit87] Witkowska A., Jablonski L., Jahn R. (2018). A convenient protocol for generating giant unilamellar vesicles containing SNARE proteins using electroformation. Sci. Rep..

[cit88] Schindelin J., Arganda-Carreras I., Frise E., Kaynig V., Longair M., Pietzsch T. (2012). *et al.*, Fiji: an open-source platform for biological-image analysis. Nat. Methods.

[cit89] Pan J., Heberle F. A., Tristram-Nagle S., Szymanski M., Koepfinger M., Katsaras J. (2012). *et al.*, Molecular structures of fluid phase phosphatidylglycerol bilayers as determined by small angle neutron and X-ray scattering. Biochim. Biophys. Acta, Biomembr..

[cit90] Lütgebaucks C., Macias-Romero C., Roke S. (2017). Characterization of the interface of binary mixed DOPC:DOPS liposomes in water: The impact of charge condensation. J. Chem. Phys..

[cit91] Lai L. Y., Samoilova R. I., Gennis R. B., Dikanov S. A. (2006). Characterization of the exchangeable protons in the immediate vicinity of the semiquinone radical at the QH site of the cytochrome *bo*_3_ from *Escherichia coli*. J. Biol. Chem..

[cit92] Choi S. K., Lin M. T., Ouyang H., Gennis R. B. (2017). Searching for the low affinity ubiquinone binding site in cytochrome *bo*_3_ from *Escherichia coli*. Biochim. Biophys. Acta, Bioenerg..

[cit93] Frericks H. L., Zhou D. H., Yap L. L., Gennis R. B., Rienstra C. M. (2006). Magic-angle spinning solid-state NMR of a 144 kDa membrane protein complex: *E. coli* cytochrome *bo*_3_ oxidase. J. Biomol. NMR.

[cit94] Deutschmann S., Rimle L., von Ballmoos C. (2021). Rapid Estimation of Membrane Protein Orientation in Liposomes. ChemBioChem.

[cit95] Lira R. B., Dimova R., Riske K. A. (2014). Giant unilamellar vesicles formed by hybrid films of agarose and lipids display altered mechanical properties. Biophys. J..

[cit96] Rideau E., Dimova R., Schwille P., Wurm F. R., Landfester K. (2018). Liposomes and polymersomes: a comparative review towards cell mimicking. Chem. Soc. Rev..

[cit97] Otrin L., Marušič N., Bednarz C., Vidaković-Koch T., Lieberwirth I., Landfester K. (2017). *et al.*, Toward Artificial Mitochondrion: Mimicking Oxidative Phosphorylation in Polymer and Hybrid Membranes. Nano Lett..

[cit98] Khan S., Li M., Muench S. P. (2016). Jeuken LJCC, Beales PA. Durable proteo-hybrid vesicles for the extended functional lifetime
of membrane proteins in bionanotechnology. Chem. Commun..

