# Supplementary figures and images for: Experimental platform for the functional investigation of membrane proteins in giant unilamellar vesicles
Source: Soft Matter. 2022 Jul 25;18(31):5877–93. doi: 10.1039/d2sm00551d (PMC9364335; doi:10.1039/d2sm00551d)

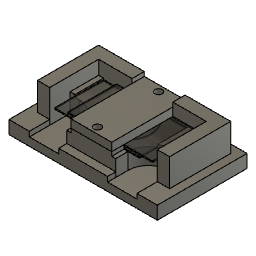

Supplement: SM-018-D2SM00551D-s001 [file SM-018-D2SM00551D-s001.f3d › FusionAssetName[Active]/Previews/small.png]
