# Supplementary material for: Experimental platform for the functional investigation of membrane proteins in giant unilamellar vesicles
Source: Soft Matter. 2022 Jul 25;18(31):5877–93. doi: 10.1039/d2sm00551d (PMC9364335; doi:10.1039/d2sm00551d)
Supplement: SM-018-D2SM00551D-s003 [file SM-018-D2SM00551D-s003.pdf]

Electronic Supplementary Material (ESI) for Soft Matter.
This journal is © The Royal Society of Chemistry 2022

Supplementary information

Experimental platform for the functional investigation of membrane proteins in giant unilamellar vesicles

Nicolas Dolder[1,*], Philipp Müller[1,*] and Christoph von Ballmoos[1]

[1]Department of Chemistry, Biochemistry and Pharmaceutical Sciences, University of Bern,

Freiestrasse 3, 3012 Bern, Switzerland

* Contributed equally to the manuscript

Correspondence: christoph.vonballmoos@unibe.ch

Supplementary Results and Discussion

GUV Formation

GUV formation by polymer assisted swelling or electroformation was compared in the absence or presence of up to 100 mM monovalent salts (sodium and potassium chloride) and 10 mM divalent salt (calcium chloride), which we consider the upper limit of physiological concentrations needed for MP investigation. GUV lamellarity was not specially investigated as previous publications show that several GUV formation methods result in the production of predominantly unilamellar vesicles, in agreement with our visual inspection, although differences between formation techniques are observed.[1–3] Thus, we focused on the analysis of the yield and size distribution of GUVs formed by the different methods. Using 20 µg lipid as starting material, GUV formation by PVA assisted swelling and electroformation, either using platinum (Pt) wires or indium-tin-oxide (ITO) coated glass slides were compared (see Experimental procedures and Figure S1 for sequence protocols and chamber designs).

As MPs are usually investigated in free-standing vesicles, yield and size distribution were characterized for carefully detached GUVs transferred from the formation chamber to 8 well chambered microscopy slides. Exemplary images of the different formation and their quantitative analysis are depicted in Figure S2 and S3. In the absence of salt, high concentrations (>7 x 10^5/ml) of GUVs were obtained for all three tested formation methods. In the presence of 100 mM monovalent salts NaCl or KCl, however, only formation via PVA assisted swelling and Pt wire formation was satisfactory. This was not unexpected, as we used an electrode distance below 2 mm in our ITO coated glass slides that was previously shown to be insufficient for GUV formation at high salt concentrations.[4] In all conditions, PVA assisted swelling yielded the highest number of GUVs in the desired size range. In the absence of salt, ~8 x 10^6 GUVs (5 – 20 µm) per 1 mL formation solution (Figure S3A) were obtained, while yields decreased 2 to 3-fold in presence of 100 mM monovalent salt. Roughly, the number of GUVs obtained from both electroformation methods with no salt was about 10 times smaller than that for PVA, yielding still sufficiently high concentrations for downstream applications. Concentrations for Pt wire formations only slightly decreased with addition of 100 mM salt. Insufficient GUV concentrations were also obtained for 10 mM calcium chloride for all three formations (Figure S2). To assess how well each formation method is suited to obtain GUVs in the desired size range, the fraction of GUVs formed with diameters of 5 - 20 µm relative to the total number was calculated (Figure S3B). The smallest fractions (<20%) were obtained for all three PVA formations, indicating a high number of small vesicles, while large fractions (~40%) were obtained for Pt wire formation in the presence of 100 mM monovalent salt and ITO formation in absence of salt. Finally, histograms (bin size = 2 µm) of the size distribution for PVA GUVs and Pt wire GUVs with and without NaCl were calculated (Figure S3C and D). In the absence of salt, PVA and Pt wire GUVs showed a similar size distribution, while a higher frequency of GUVs >10

µm was obtained for ITO formation (Figure S4). Interestingly, an increased frequency of larger GUVs (> 10 µm) is observed for Pt wire formation in the presence of NaCl compared to no salt, while PVA formation was barely affected, except for a slight increase for GUVs > 20 µm. Both NaCl and KCl behaved similarly in either PVA or Pt wire formation (Figure S5). We have used the same protocol for electroformation with and without salt and the different sizes and concentration are likely a result of the changed electrical properties of the different formation conditions.[5]

To estimate the molar yield (based on deposited lipid) and the average diameter of giant unilamellar vesicles (GUVs), we used the size distribution data and the GUV concentration obtained from each experiment as described in the Materials and Methods. The mean and standard deviation of the molar yield of free-standing GUVs and average diameter are presented in Table S1. Not surprisingly, the highest yield (~30% of deposited lipid was incorporated into GUVs) was obtained for PVA formation in the absence of salt which is similar to what has recently been reported for polymer assisted swelling using cellulose.[6] In the presence of 100 mM monovalent salt, the yield decreased to 17 - 19% for PVA formation. No influence of salt on the size was observed for PVA assisted swelling, although a study using agarose had also observed an increase in GUV diameter with increasing ionic strength.[7] All electroformations produced similar yields with around 4% under the experimental conditions used here. Unlike PVA formation, the yield was not affected by the presence of monovalent salt although a slight decrease in GUV number was observed (from ~6 x10^5 GUVs mL^{-1} in absence to ~3-4 x10^5 GUVs mL^{-1} in presence of salt in the requested size). This is explained by the larger average diameter of salt GUVs that require more lipids per GUV. Thus, the decreased concentration is balanced by the increase in frequency of large GUVs formed with Pt wire in the presence of salt, resulting in similar yields for those formations. The increased average diameter reflects the increase in frequency of large GUVs (> 10 µm) for salt GUVs (Figure S4 and S5).

HPTS leakage

Soluble fluorescent dyes are frequently used to detected substrates translocated across the lipid bilayer by membrane proteins (MPs). These dyes are usually encapsulated in GUVs and need to remain inside the vesicle for the duration of the experiment. Therefore, leakage of the dye, for example due to strong vesicle adhesion, would be detrimental for measuring MP activity. Thus, we investigated whether GUVs become permeable to the dye HPTS due to immobilization. After vesicles were left to immobilize, they were imaged by recording a Z-stack using both the Liss Rhod PE and HPTS channels. Sample images from all the different conditions tested are depicted in Figure S9. The signal from the labeled lipid Liss Rhod PE was used to identify the GUV membrane while leakage was assessed using the HPTS signal. For each vesicle, an average signal for each Z-slice was obtained which was normalized and the reciprocal was calculated to obtain values close to 0 for vesicles with HPTS leakage and larger

positive values for vesicles without leakage. To distinguish between leaky and non-leaky GUVs (Figure S7A), the maximum intensity value from the whole Z-stack (max Z intensity) was used for each vesicle and a threshold using the average median value (0.006) from GUVs at weak adhesion was defined. GUVs were thus denominated as leaky if the max Z intensity was below 25% of the median value (<0.0015). As small vesicles (< 7 µm) were recorded by only few slices and tended to be less well immobilized, they were discarded from the analysis, as were large GUVs > 20 µm which are outside of our desired size range. Figure S7B shows the distribution of max Z intensity values for PVA and Pt wire GUVs with and without 100 mM sodium chloride at two different streptavidin concentrations. Generally, the distributions are barely affected by either presence of high salt or streptavidin concentrations. Notably, however, an increased number of fully leaky GUVs is found in PVA vesicles with strong immobilization, likely due to temporary or permanent membrane rupture during the immobilization process (see below). Finally, the percentage of leaky GUVs for each of the three replicates were calculated (Figure S7C). An increase in leakage at high streptavidin density is again observed for PVA GUVs, although the error is quite large at high streptavidin density. Interestingly, GUVs formed with the Pt wire method were less affected by the increased streptavidin concentration compared to PVA GUVs. Generally, the number of leaky GUVs at weak adhesion was low.

Because we observed significant differences in the number of leaky GUVs for PVA assisted formation at 6 and 60 ng mm^{-2} streptavidin, we repeated the experiment with 0, 6, 30 and 60 ng mm^{-2} streptavidin (Figure S8A and B). Increased leakage of HPTS to the inside of GUVs was already observed at 30 ng mm^{-2} streptavidin. However, at 6 ng mm^{-2} where low leakage is still observed, PVA GUVs with 0 mM sodium chloride were poorly immobilized (20% as opposed to 100% with sodium chloride), thus a streptavidin density between 6 and 30 ng mm^{-2} should be used. In the previous experiment, we were not able to discriminate if leakage happens only during immobilization and tightness is restored or if the leakiness is permanent. This is critical, as substrates or other chemicals are added after the immobilization process. We focused on PVA GUVs due to the observed differences at various streptavidin densities and added solution containing HPTS to vesicles that were already immobilized on the slide (Figure S8C and D). Strikingly, while leakage remained low for PVA GUVs without salt (< 10%), it was highly elevated for PVA GUVs with 100 mM NaCl at strong adhesion with more than half of the vesicles showing HPTS influx.

The two different experiments (leakage during and after immobilization) show a different behavior for PVA GUVs. While no difference was observed for GUVs with and without salt for leakage during immobilization, a large difference was seen if HPTS was added to already immobilized GUVs, indicating that PVA GUVs in the absence of salt are mainly temporarily leaky during the immobilization process while PVA GUVs in the presence of salt remain leaky even after immobilization. It is important to note

that in the experiment with HTPS addition after immobilization, flow stress is exerted to the vesicles that could lead to increased HPTS permeability, especially if vesicles are unstable.

Proton leakage

One of the main interests in our group are MPs form the respiratory chain translocating protons. Thus, it is important that GUVs should be able to maintain a proton gradient. To detect passive proton leakage, GUVs containing HPTS were prepared at pH 7.4, immobilized and washed with buffer at pH 8.0, resulting in an inside acidic gradient of ΔpH=0.6. The HPTS signal was monitored for 1 h before the pH gradient was equilibrated by the addition of the protonophore gramicidin, leading to an alkalinization of the GUV lumen and an increase in HPTS ratio. The signal increase of all GUVs that were selected for analysis showed minimal increase of HPTS ratio during the 1 h incubation, which is an indication of GUV tightness towards protons (Figure S11). A low number of these GUVs displayed very small or higher values than the median of the calculated percentages, representing the outliers in Figure 3B. The latter might indicate high leakage, but we also observed some GUVs which showed only small increase after gramicidin addition, which would in turn result in a large percentage. We are not sure why these GUVs did not react to gramicidin, it is possible that they were multilamellar or that the concentration of gramicidin was not high enough to achieve efficient gramicidin incorporation in all GUVs. Further, some GUVs seem to even decrease in signal after gramicidin addition, potentially due to HPTS leakage, which might even result in negative percentages and most likely account for the outliers at very small values.

Charge-mediated fusion in an Eppendorf tube

The last step towards measurements of MPs in GUVs is the functional reconstitution of the protein. Here, we used charge-mediated fusion of positively charged small unilamellar vesicles (SUVs) to negatively charged GUVs as a method to incorporate MPs into GUVs. We started by performing experiments with empty liposomes, initiating fusion directly on the microscopy slide by addition of SUVs to immobilized GUVs. We observed a wide distribution of signals, potentially due to effects of immobilization or mixing and pipetting artifacts. To avoid these artifacts, we repeated the experiments by incubation of free-floating GUVs (approximately 0.3 – 1.6 x10^6 GUVs (5 – 20 µm) per mL) with SUVs for 15 min in an Eppendorf tube after which they were transferred into a microscopy well and left to immobilize. Z-stacks were recorded for all conditions and an average Z-projection was created for every stack. Average signal intensities for every GUV were then extracted and depicted as a signal distribution (Figure S14). Contrary to our expectations, fusion in an Eppendorf tube did not lead to a narrower signal distribution. For PVA GUVs, we observe a decrease in signal in the presence of 100 mM NaCl both for fusion in the microscopy well and in the Eppendorf tube. Interestingly, this trend was not observed for in fusion experiments with Pt-wire GUVs in the Eppendorf tube.

Supplementary Tables and Figures

Table S1: Lipid Yield and mean average diameter of GUVs prepared by PVA formation or electroformation. The lipid yield was calculated as described in Material and Methods and the mean and standard deviation of three experiments is shown. The average diameter was calculated from the diameter distributions recorded for each formation and the mean and standard deviation of the average diameters for the three experiments are shown. Values could not be determined for formations that produced insufficient GUV numbers and are labeled with ND.

	PVA		Pt wire		ITO	
	Yield [%]	Diameter [µm]	Yield [%]	Diameter [µm]	Yield [%]	Diameter [µm]
0 mM salt	31.96 ± 12.67	3.82 ± 0.16	3.69 ± 1.14	4.21 ± 0.39	4.29 ± 2.66	6.40 ± 1.36
100 mM NaCl	16.65 ± 1.86	3.81 ± 0.33	4.18 ± 0.89	7.49 ± 0.84	ND ND	ND ND
100 mM KCl	18.63 ± 9.10	3.94 ± 0.46	4.25 ± 0.46	6.48 ± 1.09	ND ND	ND ND

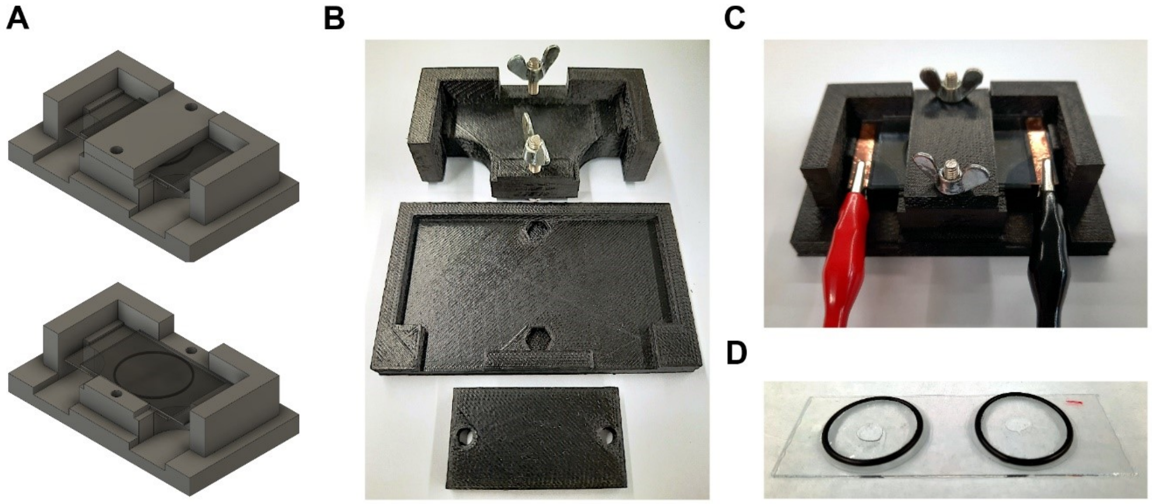

Figure S1: Assembly of the ITO electroformation and PVA formation chambers. A) 3D render of the custom ITO-coated slide holder (Supplementary File S1) with (top) and without (bottom) lid to hold the slides in place. Slides are shown with rubber O-ring as spacer. B) 3D printed parts of the slide holder. Holes are designed to fit M4 hex bolts to screw on the lid using wing nuts (not 3D printed). The holder is composed of the main holder (top), a stand (middle) and a lid (bottom). C) Assembled ITO formation chamber. ITO-coated slides are modified with adhesive copper tape and separated by a 1 mm rubber spacer. Crocodile claps are attached to the copper tape. D) PVA formation chamber. Two coverglasses (25 mm Ø) with PVA gel inside a rubber O-ring are glued to a microscopy slide for better transportation using grease for laboratories. The O-ring forms a barrier, allowing the addition of formation buffer to the gels. To protect the formation from dust, slides are covered by a lid (not shown).

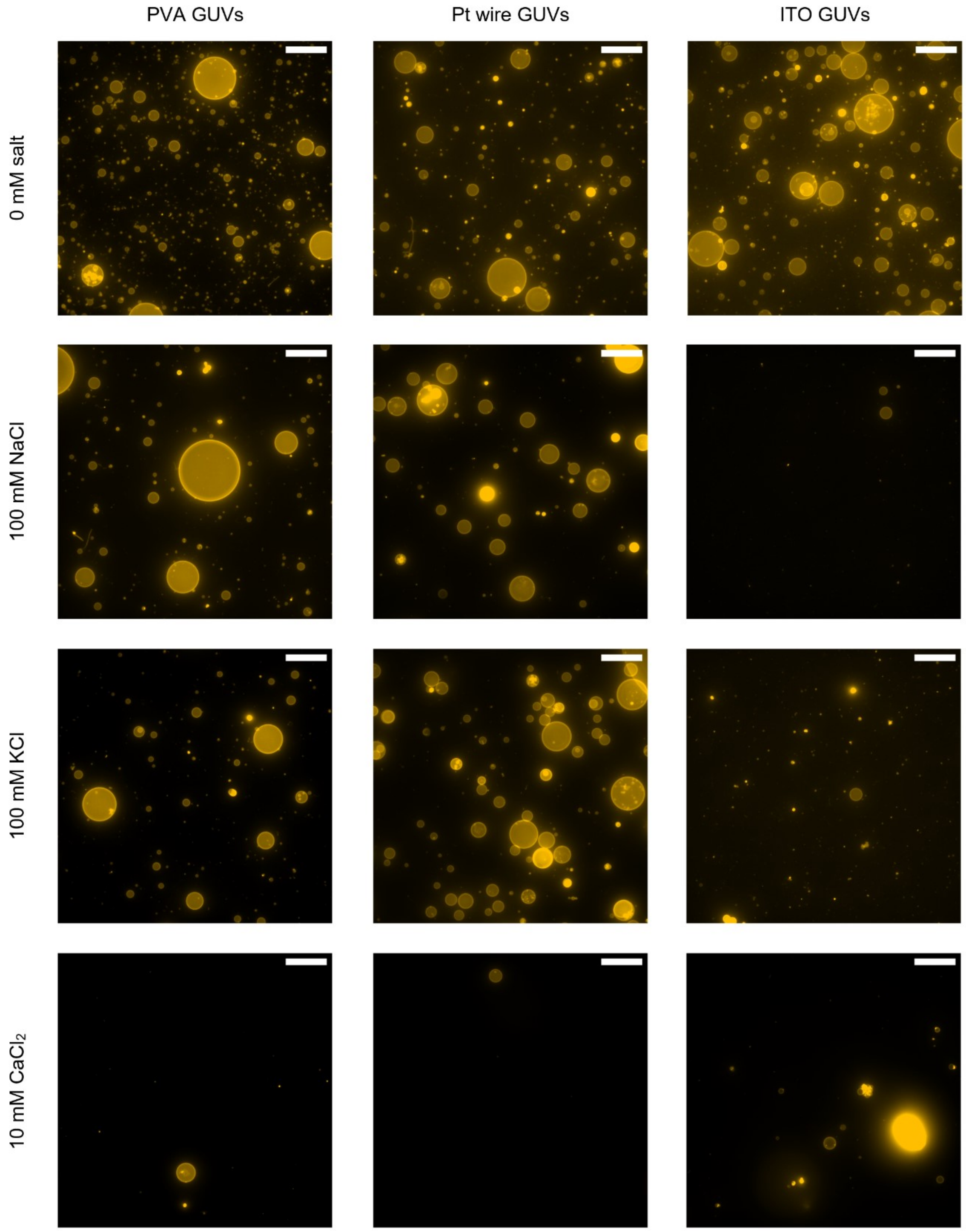

Figure S2: Representative GUV formations in the presence or absence of salt. Average Z-projections of confocal Z-stacks recorded in the Liss Rhod PE channel are shown. PVA GUV formations are shown in the first column, Pt wire formations in the second column and ITO formations in the third column. The first row depicts formations in the absence of salt, the second row with addition of 100 mM NaCl, the third row with addition of 100 mM KCl and the last row with addition of 10 mM $CaCl_2$ to the formation buffer. The scale bar is 50 µm.

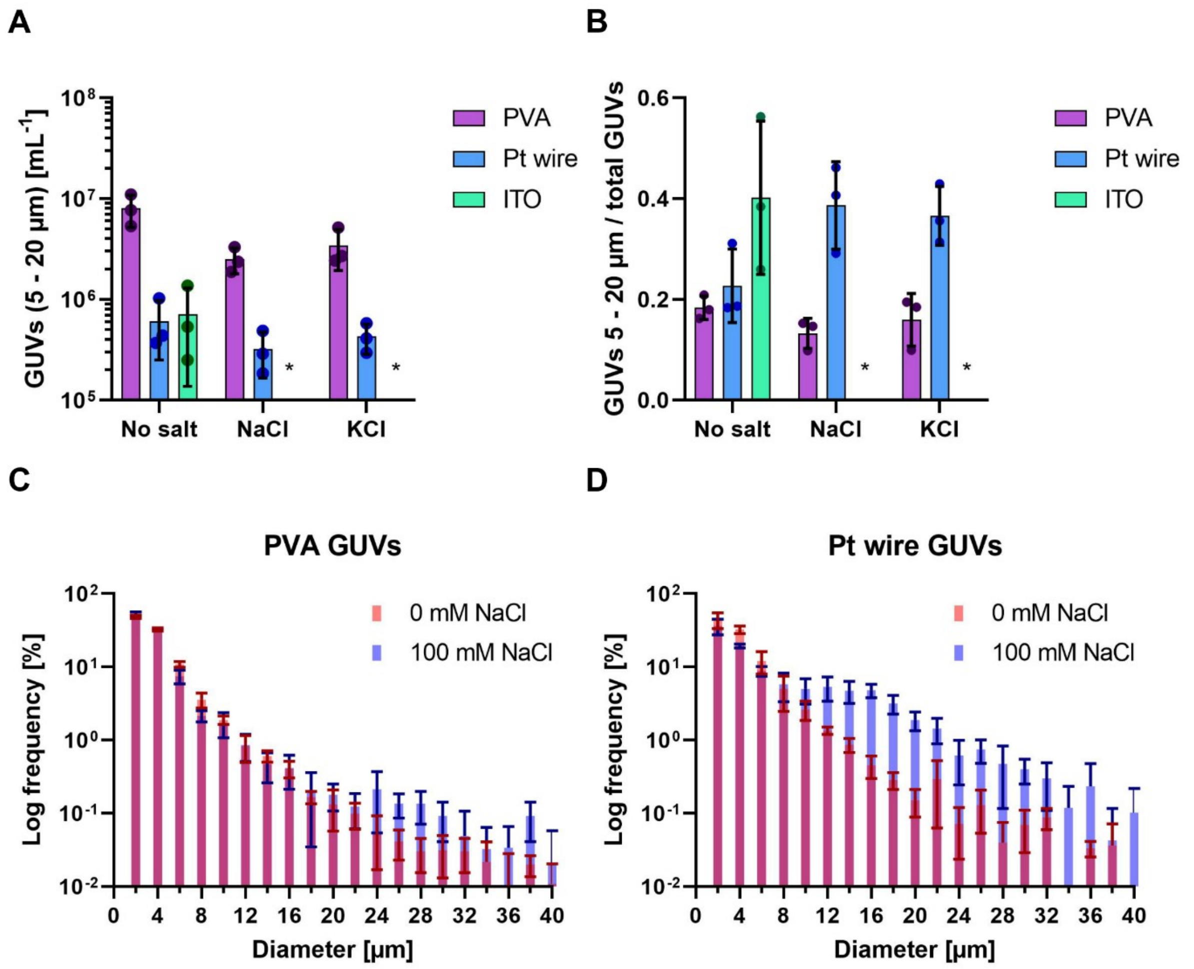

Figure S3: Characterization of GUV electroformation and PVA-assisted swelling. For each condition, size and concentration of GUVs was obtained from sampling of 9 positions in each well, conducted in three independent experiments and analyzed as previously published.[6] GUV formations that produced an insufficient number of vesicles (< 10 GUVs in the requested size range per 40x field of view) are marked by an asterisk. The height of the bar indicates the average with individual values from the experiments shown as dots. Error bars indicate the standard deviation. A) Concentration of GUVs with diameters of 5 – 20 µm in solution after removal from the formation chamber. B) Formation quality assessed by calculating the fraction of GUVs in the desired diameter range compared to the total amount of GUVs counted. C-D) Histograms of PVA formation and Pt wire formation in the presence or absence of 100 mM NaCl. Diameters were obtained from 780 – 6480 GUVs depending on the formation. The width of the bins is 2 µm and the graph is cut off at 40 µm. Frequencies are displayed on a logarithmic scale as larger vesicles have smaller frequencies. C) Histograms of GUVs prepared by PVA formation. D) Histograms of GUVs prepared by Pt wire electroformation.

Here we show the calculated histograms from the size distributions of each formation condition. A bin size of 1 µm was chosen to illustrate the size distribution in the relevant size range of 0 – 20 µm. A larger bin size of 5 µm was chosen to represent the entire size range of the formations (up to 100 µm) and the frequency is displayed in the logarithmic scale, due to the very small frequency of large GUVs.

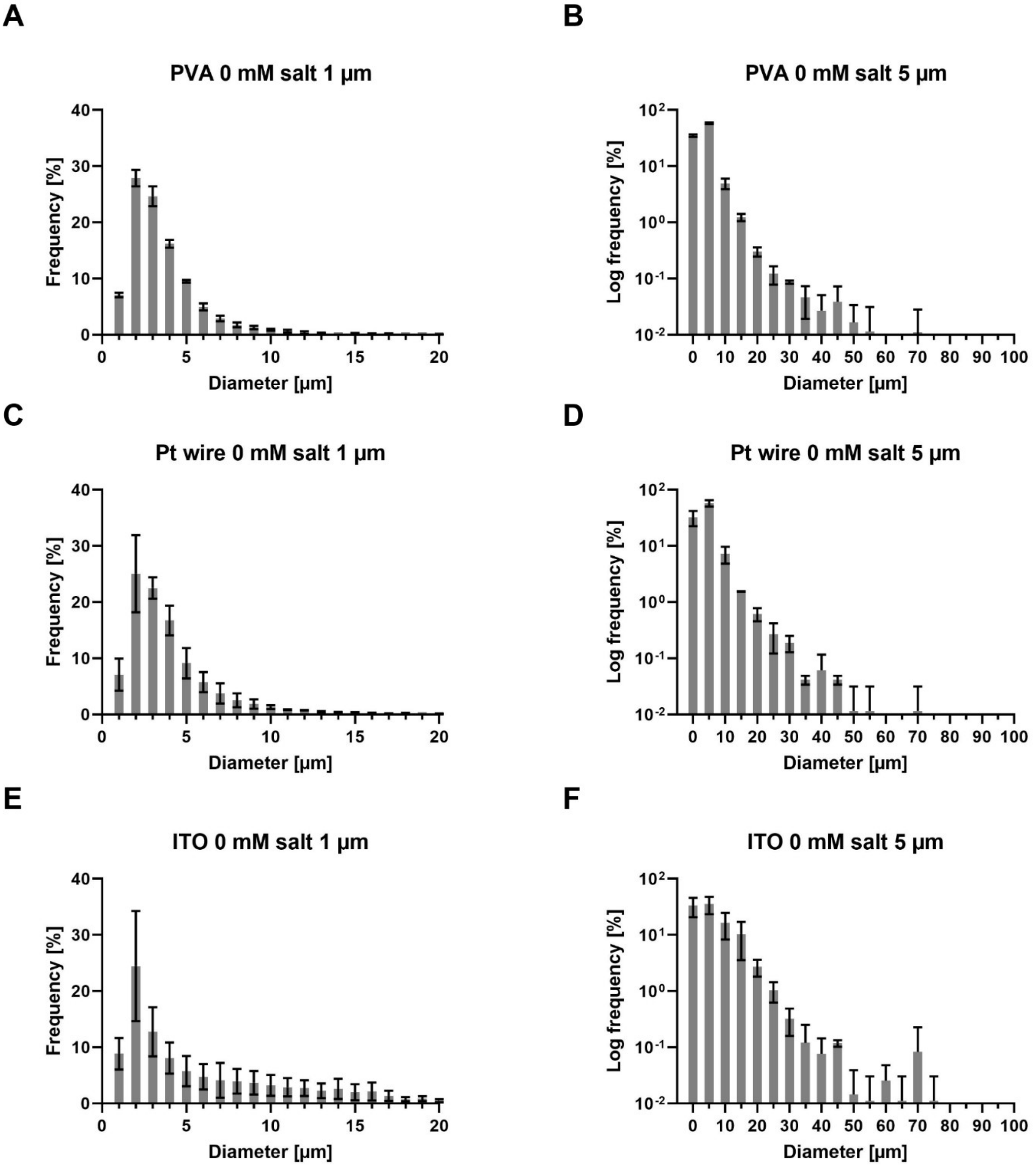

Figure S4: Histograms of GUV formations in the absence of salt. The bar height represents the average frequency and error bars the standard deviation from three experiments. A, C, E show histograms with a bin size of 1 µm cut off at 20 µm. B, D, F show histograms with a bin size of 5 µm cut off at 100 µm. The Y-axis is logarithmic as large GUVs tend to have small frequencies. A-B) Histograms

from PVA formation. C-D) Histograms from Pt wire formation. E-F) Histograms from ITO formation.

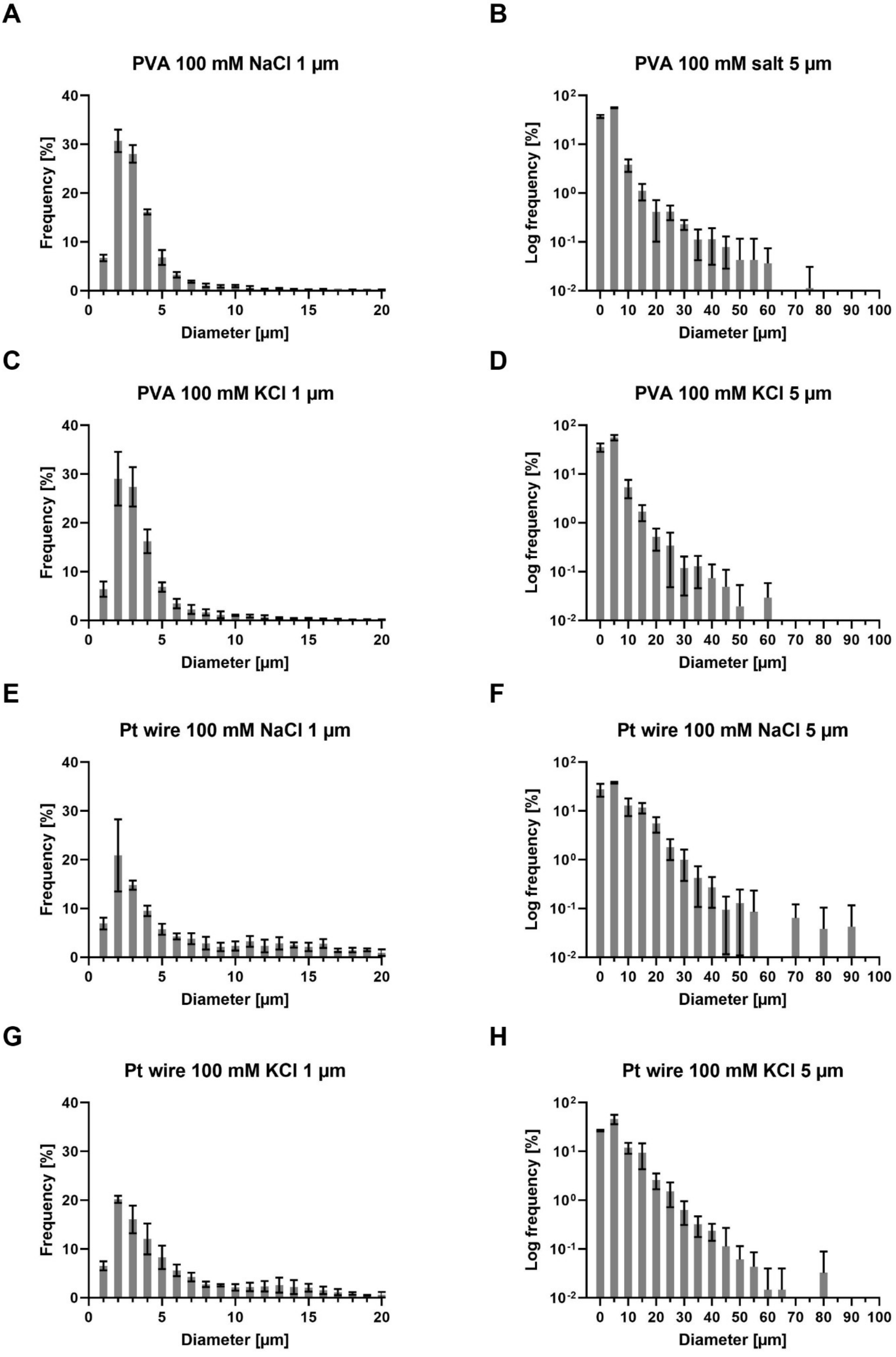

Figure S5: Histograms of PVA and Pt wire GUV formation in the presence of salt. The bar height represents the average frequency and error bars the standard deviation from three experiments. A, C, E, G show histograms with a bin width of 1 µm cut off at 20 µm. B, D, F, H show histograms with a bin width of 5 µm cut off at 100 µm. The Y-axis is logarithmic as large GUVs tend to have small frequencies. A-B) Histograms from PVA formation with 100 mM NaCl. C-D) Histograms from PVA formation with 100 mM KCl. E-F) Histograms from Pt wire formation with 100 mM NaCl. G-H) Histograms from Pt wire formations with 100 mM KCl.

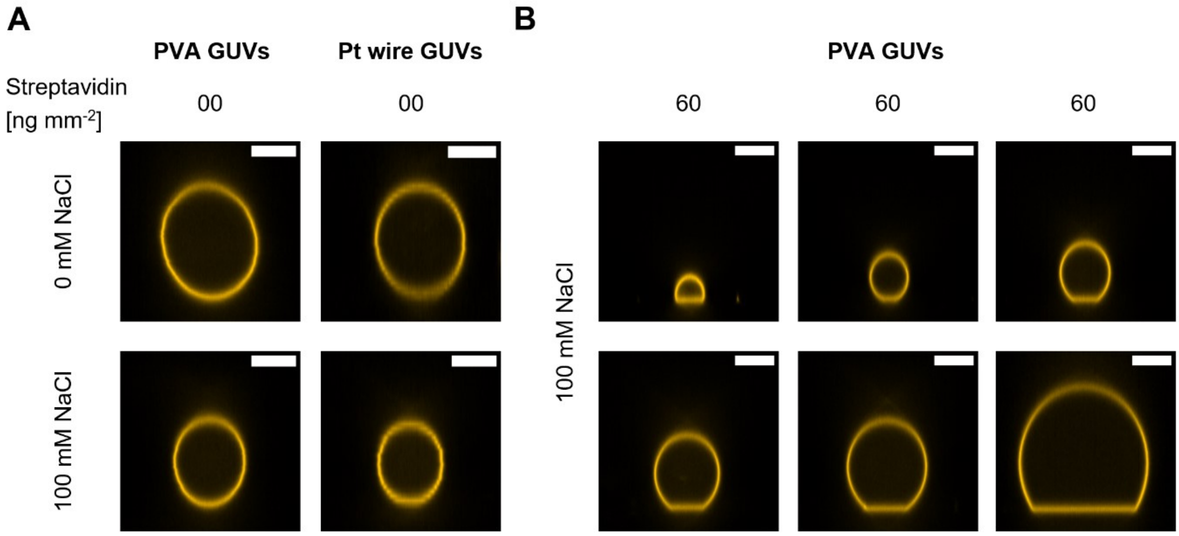

Figure S6: Spherical adhesion caps in the absence of streptavidin and heterogeneity of caps in a single well. Confocal Z-stacks were recorded and side-views of representative GUVs are shown. The scale bar is 10 µm. A) PVA and Pt wire GUVs in the presence and absence of 100 mM NaCl were immobilized at a streptavidin density of 0 ng mm^{-2} No Spherical caps are observed. B) Different PVA GUVs in the same well chamber with 100 mM NaCl immobilized at a streptavidin density of 60 ng mm^{-2} are shown.

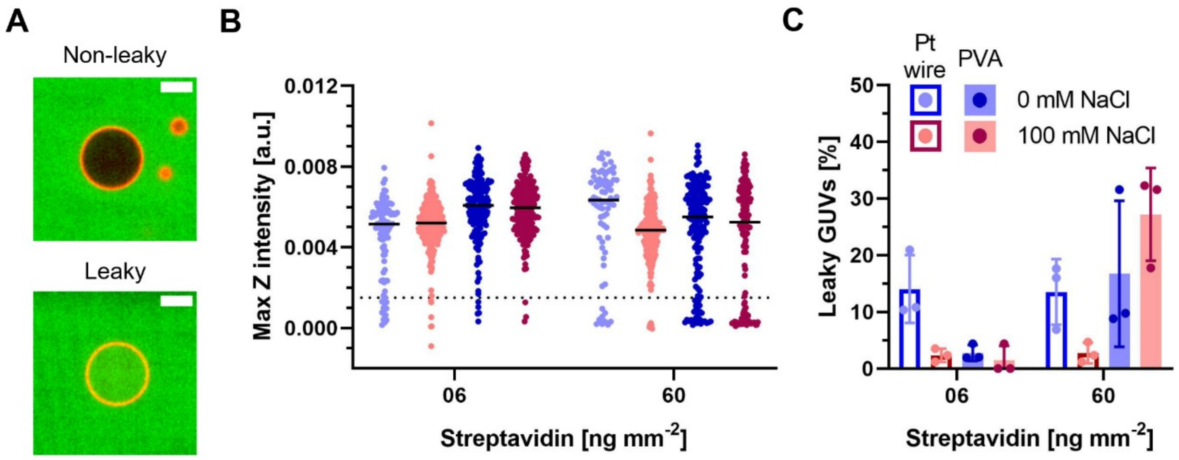

Figure S7: Immobilization-induced HPTS leakage of PVA and Pt wire GUVs (7 – 20 µm) in the presence (red) or absence (blue) of 100 mM NaCl at low and high streptavidin densities. A) Example GUV without (top) and with (bottom) HPTS leakage. Overlay of HPTS channel (green) and Liss Rhod PE channel (red). The scale bar is 10 µm. B) Combined distribution of the maximum Z intensity values from three experiments. Each dot (colors correspond to the dots in the legend of C) represents a single GUV and vesicles from three replicates are combined. The median is indicated by a black line and the threshold is depicted by a dotted line. Maximum Z intensities were extracted from Z stacks recorded in the HPTS channel according to Material and Methods. Values close to zero indicate HPTS leakage into GUVs and positive values indicate the absence of HPTS in the GUV lumen. The dotted line indicates the threshold (0.0015) used to calculate the percentage of leaky GUVs and is approximately 25% of the average median (black lines) of GUVs immobilized at a low streptavidin density. GUVs below the threshold were classified as leaky. C) Percentage of leaky GUVs from B. The height of the bar indicates the average percentage of leaky GUVs with individual values from the three experiments shown as dots. Error bars indicate the standard deviation.

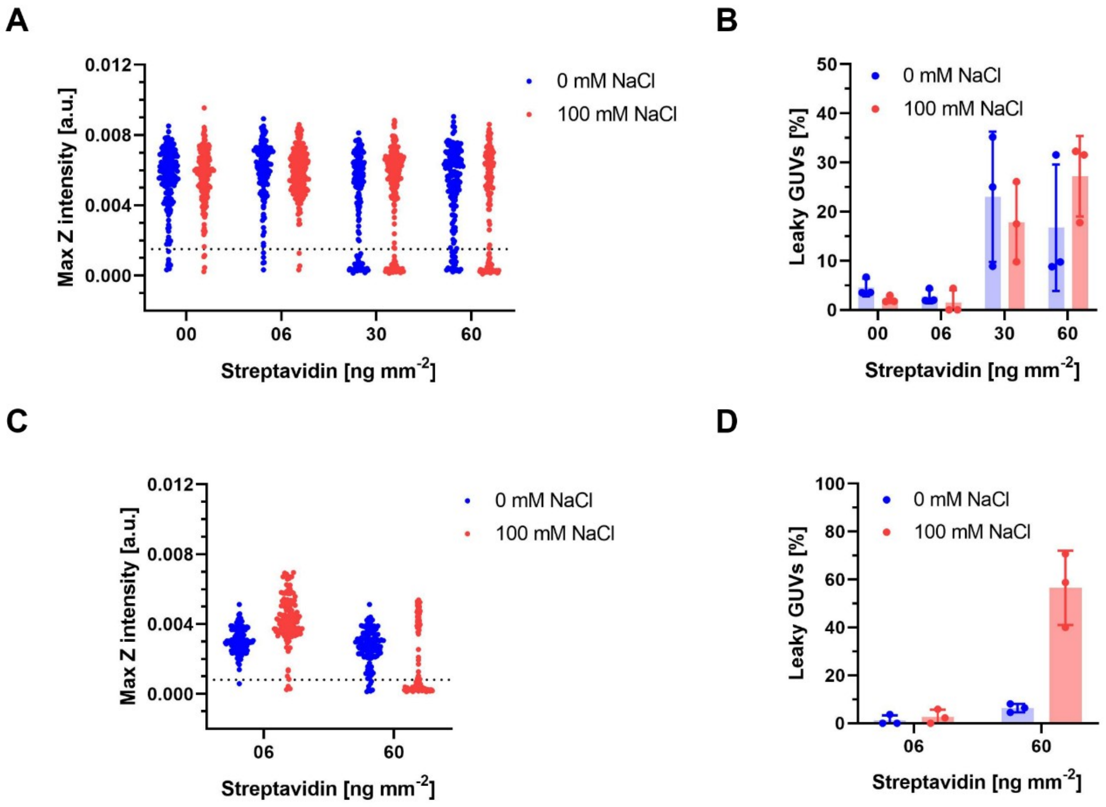

Figure S8: HPTS leakage of PVA GUVs (7 – 20 µm) in the presence (red) or absence (blue) of 100 mM NaCl at different streptavidin densities. A and B show immobilization-induced HPTS leakage at 00, 06, 30 and 60 ng mm^{-2} streptavidin. C and D show leakage of HPTS with addition of dye after immobilization at low and high streptavidin density. A and C show the combined distribution of the maximum Z intensity values from three experiments. Maximum Z intensities were extracted from Z stacks recorded in the HPTS channel according to Material and Methods. Values close to zero indicate HPTS leakage into GUVs and positive values indicate the absence of HPTS in the GUV lumen. The dotted line indicates the threshold used to calculate the percentage of leaky GUVs and is approximately 25% of the average median of GUVs immobilized at a low streptavidin densities. GUVs below the threshold were classified as leaky. A) Maximum Z intensity distribution. The threshold (0.0015) was set based on the average median at streptavidin densities of 00 and 06 ng mm^{-2}. B) Percentage of leaky GUVs from A. The height of the bar indicates the average percentage of leaky GUVs with individual values from the three experiments shown as dots. Error bars indicate the standard deviation. C) Maximum Z intensity distribution. The threshold (0.0008) was set based on the average median at a streptavidin density of 06 ng mm^{-2}. D) Percentage of leaky GUVs from C. The height of the bar indicates the average percentage of leaky GUVs with individual values from the three experiments shown as dots. Error bars indicate the standard deviation.

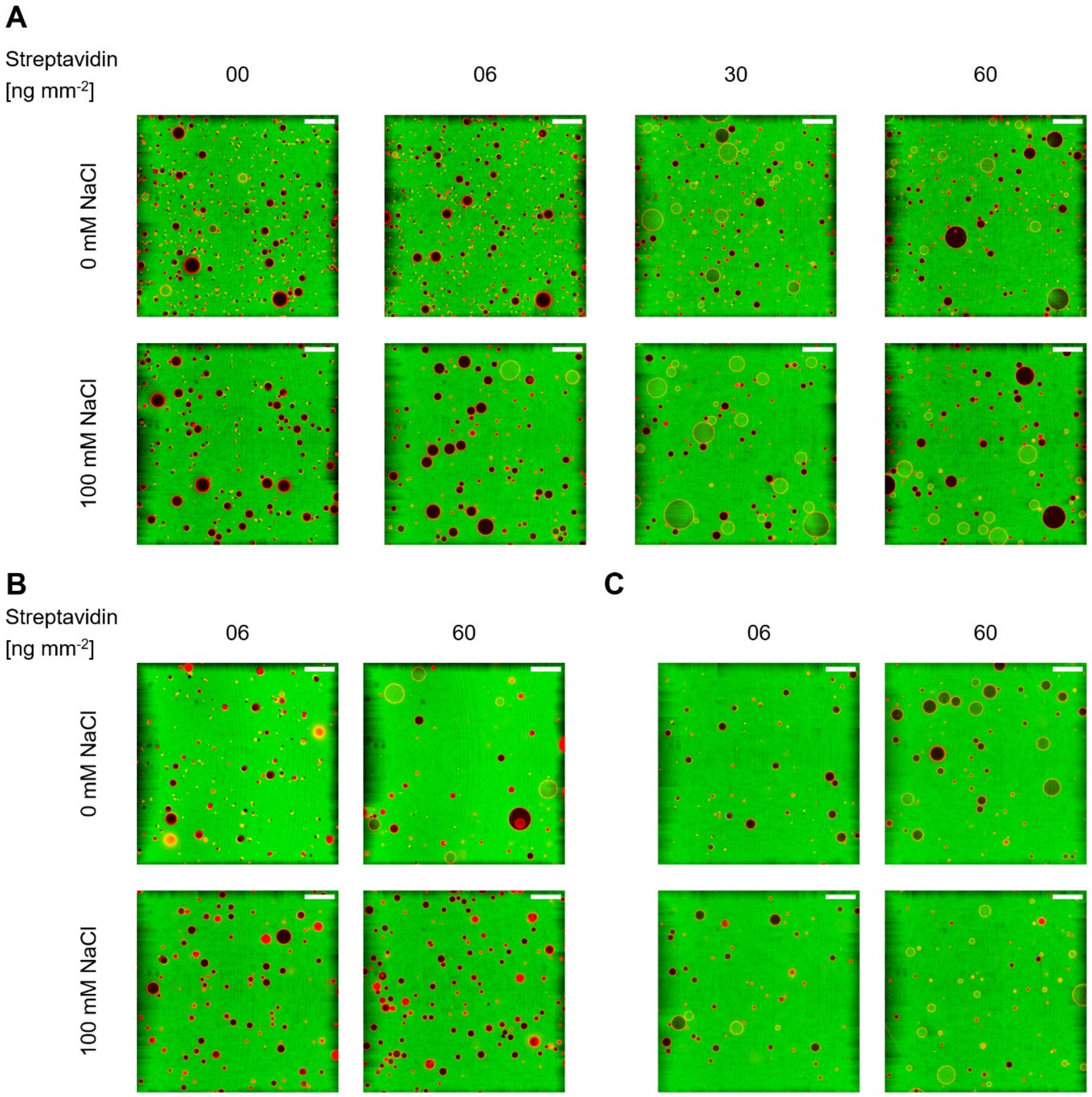

Figure S9: Confocal microscopy images of HPTS leakage in GUVs in the presence and absence of 100 mM NaCl. Overlay of HPTS channel (green) and Liss Rhod PE channel (red). Images were recorded approximately 5 µm above the slide surface. The scale bar is 50 µm. A) Images of immobilization-induced leakage of HPTS in PVA GUVs at different streptavidin densities. B) Images of immobilization-induced leakage of HPTS in Pt wire GUVs at low and high streptavidin density C) Images of leakage of HPTS in PVA GUVs with addition of dye after immobilization at low and high streptavidin density

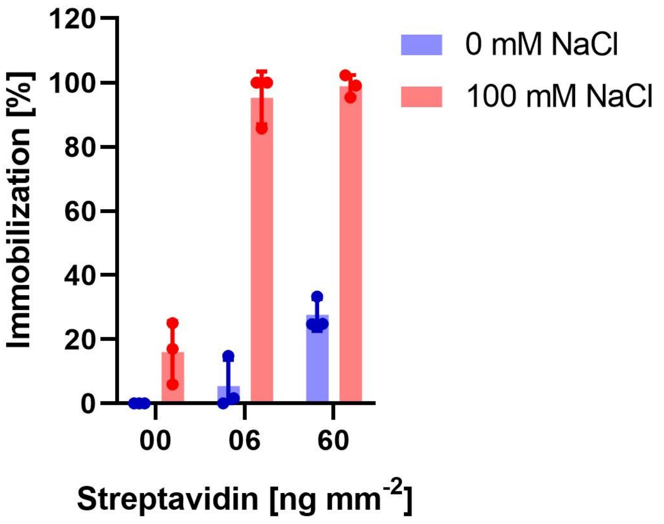

Figure S10 Immobilization assay using channel slides and PVA GUVs with 5 mM MOPS-KOH buffer. Percentage of immobilized PVA GUVs in the presence or absence of 100 mM NaCl at different streptavidin densities assessed by comparing the number of immobilized GUVs and the number of GUVs before application of flow. Data from three experiments are shown. GUVs were prepared in 5 mM MOPS-KOH pH 7.4, 200 mM sucrose with salt as indicated. The height of the bar indicates the average percentage of immobilization with individual values from the experiments shown as dots. Error bars indicate the standard deviation.

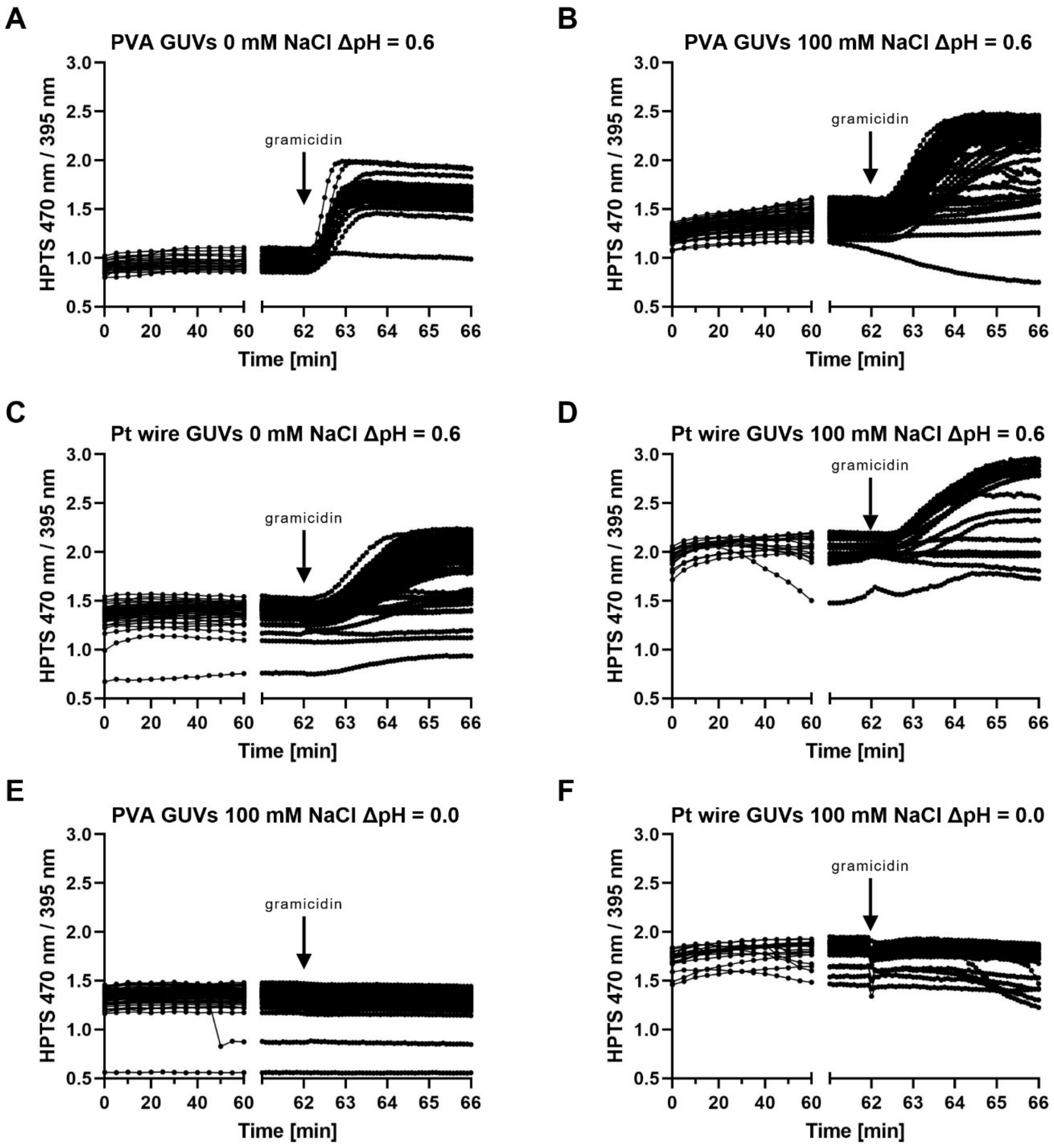

Figure S11: Proton leakage of immobilized PVA and Pt wire GUVs in the presence or absence of 100 mM NaCl. Analysis was limited to GUVs with diameters of 5 – 20 µm in the focal plane. Data are taken from a single time series for each condition. Each time point is represented by a dot. HPTS ratio of individual GUVs from one experiment are shown. Only GUVs with diameters of 5 – 20 µm in the focal plane were analyzed. A - D shows GUVs subjected to a pH gradient of 0.6 (pH inside = 7.4, pH outside = 8.0). E and F shows GUVs in the presence of 100 mM NaCl with no applied pH gradient. After 62 min, gramicidin was added to equilibrate the inner and outer pH. A) PVA GUVs in the absence and B) in the presence of 100 mM NaCl. C) Pt wire GUVs in the absence and D) in the presence of 100 mM NaCl. E) PVA GUVs and F) Pt wire GUVs without pH gradient.

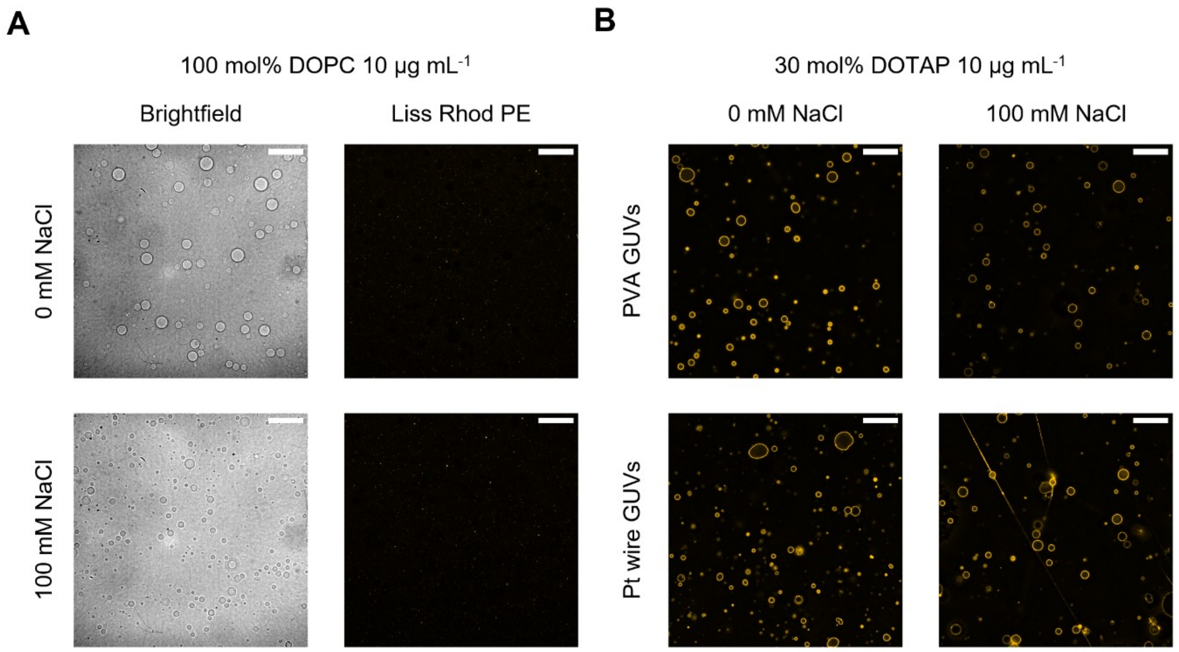

Figure S12: Representative confocal microscopy images of charge-mediated fusion performed in an 8 well chambered slide between negatively charged GUVs and neutral or positively charged SUV. The scale bar is 50 µm. All images from each channel were processed identically. A) Charge-mediated fusion with PVA GUVs and neutral SUVs (10 µg mL^{-1}) in the presence or absence of 100 mM NaCl. Brightfield and Liss Rhod PE images are shown. No fusion was observed, indicated by the absence of visible GUVs in the Liss Rhod PE channel. B) Charge-mediated fusion with PVA and Pt wire GUVs and positively charged SUVs (10 µg mL^{-1}) in the presence or absence of 100 mM NaCl.

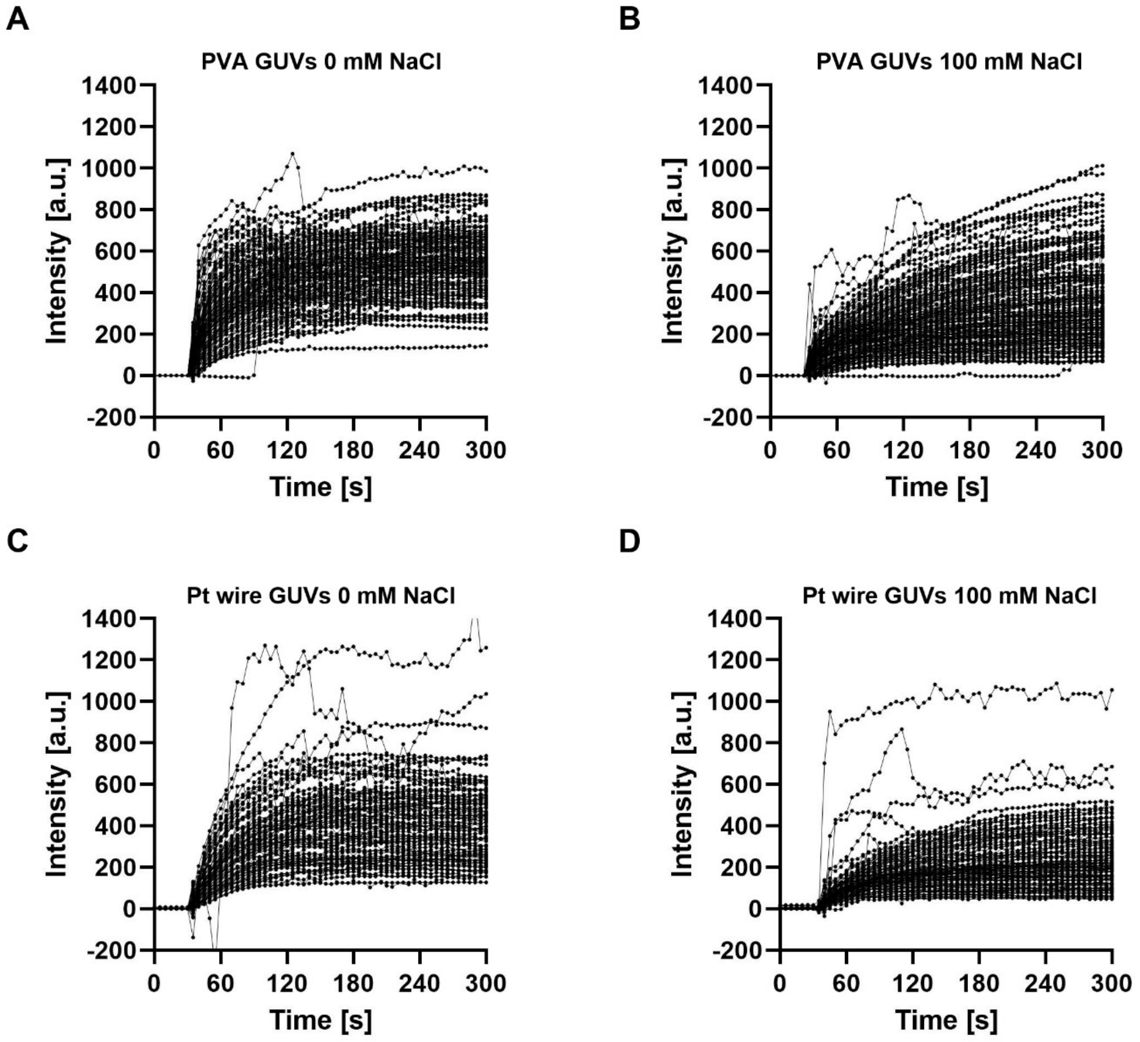

Figure S13: Charge-mediated fusion performed in an 8 well chambered slide between negatively charged GUVs and positively charged SUVs (10 µg mL^{-1}). Combined traces of GUVs from four experiments are shown. Only GUVs with diameters of 5 – 20 µm in the focal plane were analyzed (90 – 170 GUVs for all four experiments). Each recorded time point is represented as a dot. A) Fusion with PVA GUVs in the absence and B) in the presence of 100 mM NaCl. C) Fusion with Pt wire GUVs in the absence and D) in the presence of 100 mM NaCl.

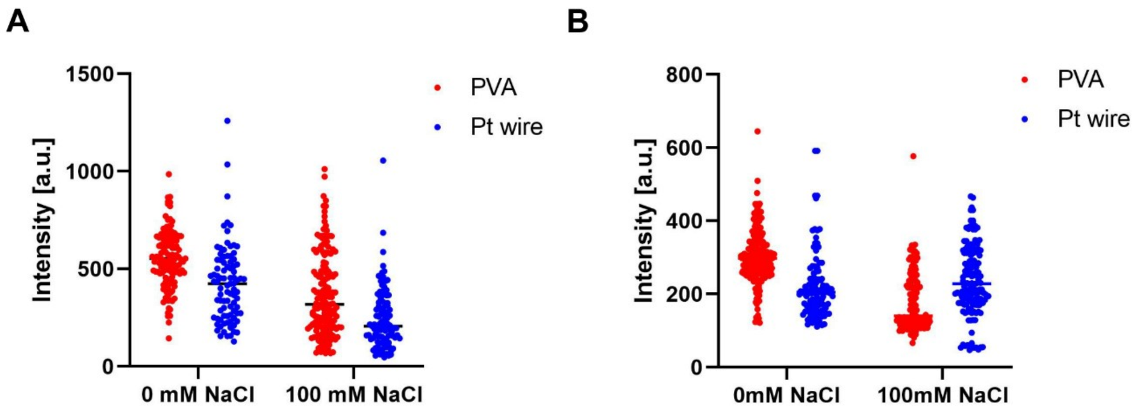

Figure S14: Charge-mediated fusion between negatively charged GUVs and positively charged SUVs. Combined distribution of Liss Rhod PE intensities from four experiments for PVA and Pt wire GUVs in the presence or absence of 100 mM NaCl. A) Intensity distribution from a single imaging plane after 270 s of fusion in an 8 well chambered slide. Concentration of SUVs in the slide was 10 µg mL^{-1}. B) Intensity distribution from an average projection of a Z-stack after fusion in an Eppendorf tube. Concentration of SUVs in the tube was 10 µg mL^{-1}. GUVs were fused for 15 min in the tube, added to an 8 well chambered slide coated with BSA and imaged after they were left to settle for 1h.

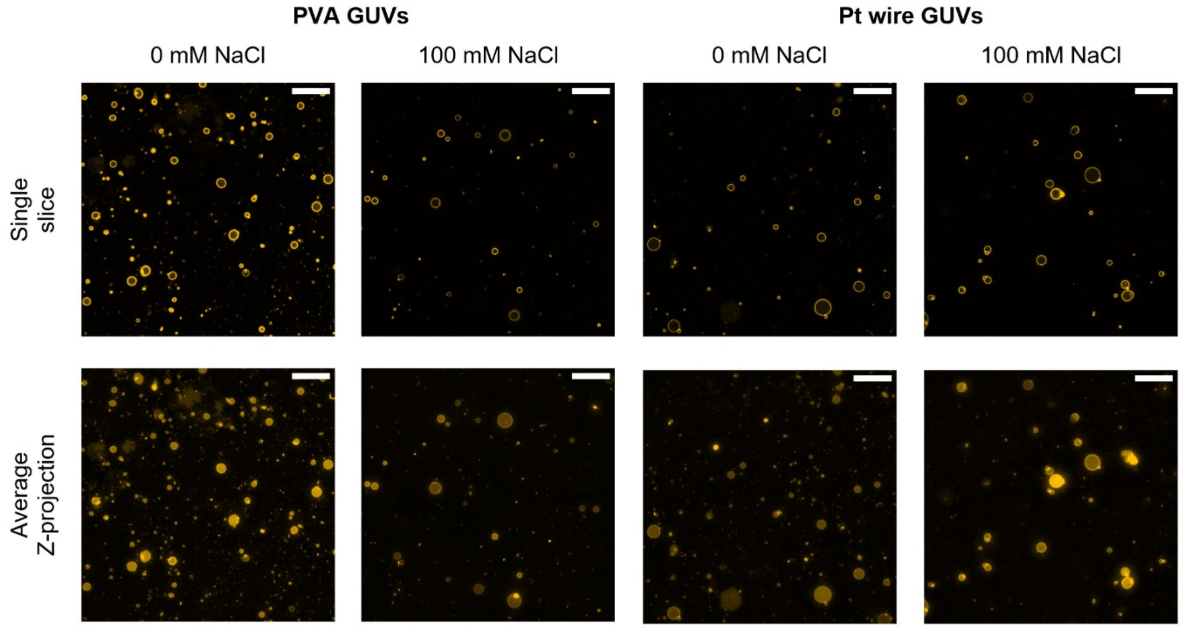

Figure S15: Representative confocal microscopy images of charge-mediated fusion between negatively charged GUVs and positively charged SUV performed in an Eppendorf tube. A single slice and the average Z-projection from a Z-stack image are shown for fusion with PVA and Pt wire GUVs and positively charged SUVs in the presence or absence of 100 mM NaCl. Concentration of SUVs in the tube was 10 µg mL^{-1}. GUVs were fused for 15 min in the tube, added to an 8 well chambered slide

coated with BSA and imaged after they were left to settle for 1h. The scale bar is 50 µm. All single slice images were processed identically. All average Z-projections were processed identically.

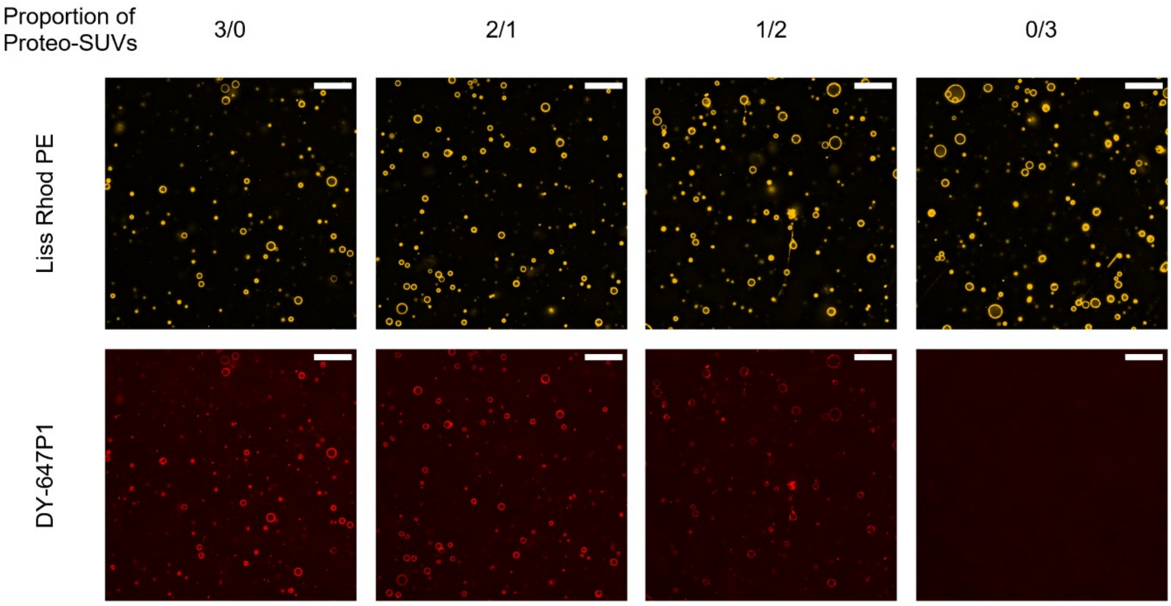

Figure S16: Confocal microscopy images of PVA GUVs at 0 mM NaCl fused with empty and proteo-SUVs (final concentration 40 µg mL^{-1}) containing DY-647P1-labeled cytochrome *bo$_3$* ubiquinol oxidase. Empty and proteo-SUVs were mixed at different ratios with proteo-SUV proportions of 1, 2/3, 1/3 and 0. Both the Liss Rhod PE and DY-647P1 channel are depicted. The scale bar is 50 µm. All images from each channel were processed identically.

Supplementary Movie S1: Charge-mediated fusion of rhodamine labeled positively charged empty SUVs with negatively charged GUVs in the microscopy well using 10 mM MOPS-BTP pH 7.4, 100 mM NaCl, and 200 mM glucose. A time series was recorded for 60 s with 1 s intervals using a 40x objective (scale bar 50 µm). The movie is displayed at a rate of 5 frames per second. SUVs were added after 6 seconds.

Supplementary File S1: The Autodesk Fusion 360 Archive File (*.f3d) of the slide holder used for ITO electroformation.

References

1. Akashi, K. I., Miyata, H., Itoh, H. & Kinosita, K. Preparation of giant liposomes in physiological conditions and their characterization under an optical microscope. *Biophys. J.* **71**, 3242–3250 (1996).
2. Chiba, M., Miyazaki, M. & Ishiwata, S. Quantitative analysis of the lamellarity of giant liposomes prepared by the inverted emulsion method. *Biophys. J.* **107**, 346–354 (2014).
3. Parigoris, E. *et al.* Facile generation of giant unilamellar vesicles using polyacrylamide gels. *Sci. Rep.* **10**, 1–10 (2020).
4. Lefrançois, P., Goudeau, B. & Arbault, S. Electroformation of phospholipid giant unilamellar vesicles in physiological phosphate buffer. *Integr. Biol. (United Kingdom)* **10**, 429–434 (2018).
5. Ghellab, S. E., Mu, W., Li, Q. & Han, X. Prediction of the size of electroformed giant unilamellar vesicle using response surface methodology. *Biophys. Chem.* **253**, 106217 (2019).
6. Pazzi, J. & Subramaniam, A. B. Nanoscale Curvature Promotes High Yield Spontaneous Formation of Cell-Mimetic Giant Vesicles on Nanocellulose Paper. *ACS Appl. Mater. Interfaces* **12**, 56549–56561 (2020).
7. Peruzzi, J., Gutierrez, M. G., Mansfield, K. & Malmstadt, N. Dynamics of Hydrogel-Assisted Giant Unilamellar Vesicle Formation from Unsaturated Lipid Systems. *Langmuir* **32**, 12702–12709 (2016).
